# The Antioxidant Activity of Quercetin in Water Solution

**DOI:** 10.3390/biomimetics2030009

**Published:** 2017-06-27

**Authors:** Riccardo Amorati, Andrea Baschieri, Adam Cowden, Luca Valgimigli

**Affiliations:** 1 University of Bologna, Department of Chemistry “G. Ciamician”, Via S. Giacomo 11, 40126 Bologna, Italy; andrea.baschieri2@unibo.it; 2 School of Chemistry (Rm 267), University of Edinburgh, West Mains Road, Edinburgh EH9 3FJ, UK; adamcowden@gmail.com

**Keywords:** catechol, peroxyl radicals, proton-coupled electron transfer, kinetics, thermodynamics, mechanisms

## Abstract

Despite its importance, little is known about the absolute performance and the mechanism for quercetin’s antioxidant activity in water solution. We have investigated this aspect by combining differential oxygen-uptake kinetic measurements and B3LYP/6311+g (d,p) calculations. At pH = 2.1 (30 °C), quercetin had modest activity (*k*_inh_ = 4.0 × 10^3^ M^−1^ s^−1^), superimposable to catechol. On raising the pH to 7.4, reactivity was boosted 40-fold, trapping two peroxyl radicals in the chromen-4-one core and two in the catechol with *k*_inh_ of 1.6 × 10^5^ and 7.0 × 10^4^ M^−1^ s^−1^. Reaction occurs from the equilibrating mono-anions in positions 4′ and 7 and involves firstly the OH in position 3, having bond dissociation enthalpies of 75.0 and 78.7 kcal/mol, respectively, for the two anions. Reaction proceeds by a combination of proton-coupled electron-transfer mechanisms: electron–proton transfer (EPT) and sequential proton loss electron transfer (SPLET). Our results help rationalize quercetin’s reactivity with peroxyl radicals and its importance under biomimetic settings, to act as a nutritional antioxidant.

## 1. Introduction

**Scheme 1:**
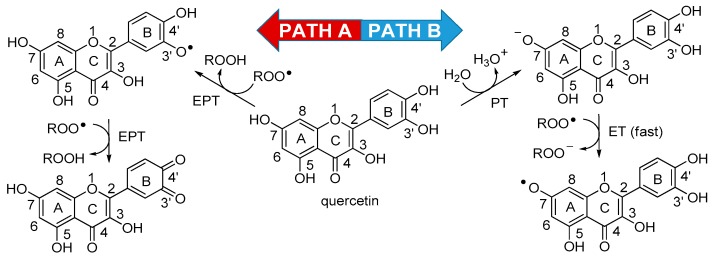
Possible reaction pathways for quercetin in water. Two mechanistic possibilities, arising from current literature, could account for the antioxidant activity of quercetin in water solution: Path A illustrates the electron–proton transfer (EPT) to peroxyl radicals from the catechol moiety, while Path B depicts the sequential proton loss electron transfer (SPLET = PT/ET) to water/peroxyl radicals. ET: Electron transfer; PT: Proton transfer.

Quercetin is perhaps the most famous flavonoid antioxidant: a member of the flavonols family, bearing a catechol moiety (ring B) linked in position 2 to the polyhydroxylated chromen-4-one core (Scheme 1). It is found in a large variety of dietary vegetables [1], which makes its presence in raw food nearly ubiquitous, and the typical daily intake was estimated as 25 mg/person/day in the U.S. diet [2]. Some sources such as capers, dill, cilantro, radish, carob, fennel, radicchio and onions are particularly rich, with 30–230 mg of quercetin or its glycosides per 100 g of edible portion [1,2,3,4,5], while green and black tea infusions have been reported to contain, respectively, about 480 and 330 mg/L of quercetin glycosides [1]. Considering that oral bioavailability can be as large as 50% (including its phenolic metabolites) [1,3], clearly, under a vegetable-rich dietary regime, quercetin can significantly contribute to the physiological antioxidant defense. Quercetin has also been attributed a range of healthy biological activities, including anti-inflammatory, blood vessel protecting, anti-platelet aggregation, antiviral, anti-cataract, enhancement of cognitive function, and anti-cancer [1,2,6,7,8,9]. Although regulatory agencies such as the European Food Safety Authority (EFSA) indicate that there is insufficient clinical evidence to support them [10], it is noteworthy that many such health-related claims are directly or indirectly associated to quercetin’s purported antioxidant activity. Its antioxidant activity has also recently been exploited, for instance, in the development of bioinspired synergic nano-antioxidants [11], or of co-delivery vincristine–quercetin nanodrugs for the treatment of lymphoma [12], and it has inspired the design of pH-responsive nanocarriers for drug release [13]. Owing to its importance, the antioxidant activity of quercetin has been the subject of many investigations [14,15]; however, surprisingly little is known about the actual mechanism and relevant quantitative values of its antioxidant activity under biomimetic settings (i.e., on the mechanism and absolute kinetics of reaction with peroxyl radicals [16,17,18,19,20] in water). 

At variance with the assessment of antioxidant activity by rapid assays [20], which has occurred in the majority of studies, and carries no mechanistic information [20], relevant kinetic data on the reaction with peroxyl radicals—namely the absolute rate constant and the stoichiometric factor for the antioxidant, *k*_inh_ and *n*, respectively—have recently been obtained by inhibited autoxidation studies in organic solution, specifically in chlorobenzene [11,21,22,23], in *tert*-butanol [21] and in acetonitrile [11,23]. Those studies converge, indicating that the catechol ring B is the “active” moiety, trapping *n* = 2 peroxyl radicals by formal H-atom transfer (the mechanism can actually be described as a concerted electron–proton transfer, EPT [24]) as depicted in Scheme 1, Path A. The values of *k*_inh_ recorded in those studies range ~5 × 10^5^ M^−1^ s^−1^ in PhCl (30 °C), ~2 × 10^4^ M^−1^ s^−1^ in *t*-BuOH (50 °C) to ~1 × 10^4^ M^−1^ s^−1^ in MeCN (30 °C), being superimposable to those of catechol itself [22] (See Table 1), and indicating that the polyhydroxy chromen-4-one core has little role in the antioxidant activity. On the other hand, using the persistent 2,2-diphenyl-1-picrylhydrazyl radical (DPPH^•^) as the model oxidant, Litwinienko and coworkers found that in protic solvents the reactivity of several flavonoids, including quercetin, was related to their p*K* for acid dissociation (p*K*_a_). Reaction of quercetin was found to be 1000-fold faster in methanol than in dioxane and was suggested to occur upon deprotonation in position 7 (ring A), via a mechanism named sequential proton loss electron transfer (SPLET) [25], and composed of a proton transfer (PT) to the solvent and an electron transfer (ET) to the oxidizing radical, as depicted in Scheme 1, Path B. This indicates that, owing to its acidity, in water solution at pH 7.4, quercetin should react with peroxyl radicals by a similar mechanism, in which the catechol has only a secondary role. Unfortunately, the study of the antioxidant behavior of water-soluble quercetin in water solution has so far been precluded by the lack of suitable methods of investigation. Our recent development and validation of one such method [26] has paved the way to filling this fundamental gap. Therefore, we report here a kinetic study on the antioxidant behavior of quercetin in water solution, accompanied with quanto-mechanical calculations aimed at clarifying its mechanism of reaction with peroxyl radicals. Calculations have also been matched with previously available spectroscopic data, so to compose a hopefully clear picture of the redox chemistry of quercetin in water, under biomimetic settings. 

## 2. Materials and Methods

### 2.1. Materials

All chemicals and solvents were of the highest purity commercially available (Sigma–Aldrich, Milan, Italy). 2,2′-Azobis(2-methylpropion-amidine)dihydrochloride (AAPH) and quercetin were used as received. 2,2,5,7,8-Pentamethyl-6-chromanol (PMHC) was recrystallized from hexane, and catechol was recrystallized from ethyl acetate/hexane. Tetrahydrofuran (THF) was distilled and stored under argon at 5 °C; the content in hydroperoxides was determined by spectrophotometry at 262 nm in isopropanol upon reaction with triphenylphosphine, and found to be <50 ppm (µg g^−1^) [26]. Buffers were prepared in bidistilled water as previously described [26]: buffer pH 2.1, NaH_2_PO_4_·2H_2_O (0.39 g, 0.05 mole) and H_3_PO_4_ 85% (0.17 mL, 0.05 mole) were dissolved in water (50 mL); buffer pH 7.4, Na_2_HPO_4_ (0.595 g, 0.096 mole) and NaH_2_PO_4_·2H_2_O (0.125 g, 0.016 mole) were dissolved in water (50 mL). Buffer solutions were mixed with the desired amount of THF (typically 3:1 *v*/*v*) after having adjusted the pH to the desired value [26].

### 2.2. Kinetic Measurements

Autoxidation experiments were performed in a two-channel oxygen-uptake apparatus based on a Validyne DP 15 differential pressure transducer (Validyne Engineering, Northridge, CA, USA) built in our laboratory and described previously [27]. Azo-initiator AAPH was prepared in concentrated stock solutions that were injected into the reaction mixture to the desired final concentrations (typically 12.5–75 mM). AAPH solutions were freshly prepared every 4 h and stored at 5 °C to avoid excessive hydrolysis. In a typical experiment, an air-saturated solution of THF–water (1:3 *v*/*v*) containing the desired buffer (0.1 M) and the initiator was equilibrated with an identical reference solution containing an excess of PMHC. After equilibration, and when a constant O_2_ consumption was reached, a concentrated solution of the antioxidant was injected into the sample flask. The oxygen consumption of the sample was measured, after calibration of the apparatus, from the differential pressure recorded with time between the two channels. Initiation rates, *R*_i_, were determined for each set of conditions by matching autoxidation experiments, using PMHC as the reference antioxidant, by means of Equation (1). Absolute *k*_inh_ values were determined, after independent assessment of *R*_i_, from 4 to 10 inhibited autoxidation experiments with antioxidant (AH) concentration in the range 10–50 µM by means of Equation (2) and values of *n* were determined from the same experiments by Equation (1) [26,27].

(1) $$R_{i}=\frac{n\left[\mathrm{Antioxidant}\right]}{\tau }$$

(2) $$-\frac{d\left[O_{2}\right]}{dt}=\frac{k_{p}\left[\mathrm{THF}\right]R_{i}}{nk_{\mathrm{inh}}\left[\mathrm{AH}\right]}$$

### 2.3. Calculations

Geometry optimization and frequencies were computed in the gas phase at the B3LYP/6-31+g (d,p) level; stationary points were confirmed by checking the absence of imaginary frequencies. Thermochemistry was computed at 298 K using the scaling factor of 0.9806 [28]. Relative stabilities in the gas phase and in water were computed by applying the free energy correction at 298 K to single point calculations at the B3LYP/6-311+g (d,p) level without and with the polarizable continuum model (PCM), respectively. Bond dissociation enthalpies (BDE_OH_) values were computed by the isodesmic approach using unsubstituted phenol as the reference, whose BDE_OH_ in water is 88.2 kcal/mol, by using enthalpy corrected total free energy in solution obtained by the PCM method [29,30]. The ultraviolet–visible (UV–vis) spectra of the most stable conformers were calculated by time-dependent density functional theory (TD-DFT), performed at the B3LYP/6-311+g (d,p) level either in the gas phase or in water by using the PCM model [31]. Conformational isomers had very similar calculated transitions in the wavelength range considered (350–700 nm), therefore only the most stable conformers were considered. Calculations were performed by using Gaussian 03 software [32] (see Appendix A in the Supplementary Materials). To visually compare calculated spectra with experimental spectra, each transition obtained by TD-DFT calculation was convoluted by a Gaussian function *g_i_* (*x*), Equation (3), where *e_i_*, *fw_i_* and *σ_i_* are the energy (in eV), the oscillator strength and the full width at half maximum (FWHM) of the peak, respectively.

(3) $$g_{i}(x)=2\sqrt{\frac{\ln2}{\pi }}\frac{fw_{i}}{\sigma_{i}}\exp\left(-{\left(\frac{2\left(x-e_{i}\right)\sqrt{\ln2}}{\sigma_{i}}\right)}^{2}\right)$$

The UV–vis spectrum *f*(*x*) is built as a sum of *N* bands (Equation (4)), where *S* is a scale factor and *g_i_* (*x*) is the Gaussian function defined above.

(4) $$f(x)=S\sum_{i=1}^{N}g_{i}(x)$$

The scale factor *S* was adjusted manually, on a trial-and-error basis, to reproduce the intensity of experimental spectra, whereas *σ_i_* was fixed to 0.5 eV. Calculated spectra were finally converted to the wavelength scale (nm) to be compared to experimental spectra [33]. 

## 3. Results and Discussion

### 3.1. Kinetic Measurements with Peroxyl Radicals

**Figure 1:**
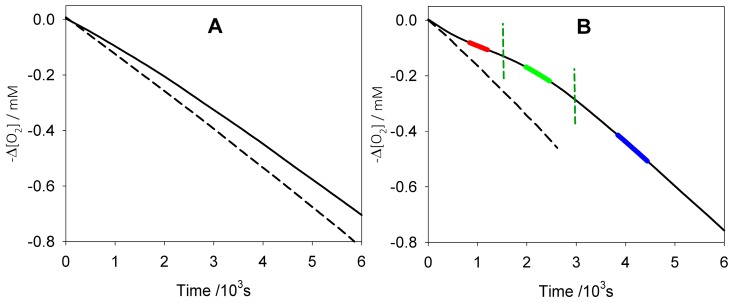
Oxygen consumption during the autoxidation of tetrahydrofuran (THF, 3.1 M) initiated by 2,2′-azobis(2-methylpropion-amidine)dihydrochloride (AAPH, 0.025 M) at 30 °C without inhibitors (dash) or in the presence of quercetin (solid). (**A**) In phosphate buffer 0.1 M pH = 2.1 with quercetin 2.5 × 10^−5^ M; (**B**) In phosphate buffer 0.1 M pH = 7.4 with quercetin 5.0 × 10^−6^ M, vertical lines indicate the time lapse corresponding to the trapping of *n* = 2 peroxyl radicals.

The antioxidant activity of quercetin was measured by studying the inhibited autoxidation of THF in buffered water solution initiated at 30 °C by the thermal decomposition of azo-initiator AAPH [26], monitoring oxygen consumption by a differential oxygen-uptake apparatus [26,27] (Figure 1). PMHC, a less lipophilic mimic of α-tocopherol with identical reactivity to peroxyl radicals [26], was used as the reference antioxidant. Measurements were performed both at pH 2.1 and at pH 7.4 and results are collected in Table 1 along with those of reference antioxidants obtained under comparable settings.

At pH 2.1, quercetin exhibited modest antioxidant behavior: at concentrations up to 50 µM, it was only able to slow down the oxygen uptake, without giving a neat inhibition period as observed with reference PMHC, thereby preventing the assessment of *n*, the number of peroxyl radicals trapped by each antioxidant molecule. The measured rate constant *k*_inh_, calculated by assuming *n* = 2, was 4.0 × 10^3^ M^−1^ s^−1^, very similar to that recorded for simple catechol. This result adds to kinetic measurements in organic solvents (Table 1), showing matched reactivity of quercetin and catechol, and suggesting a superimposable reaction mechanism: a rate-determining concerted EPT from the catechol moiety to the peroxyl radical (Scheme 1, Path A).

**Scheme 2:**
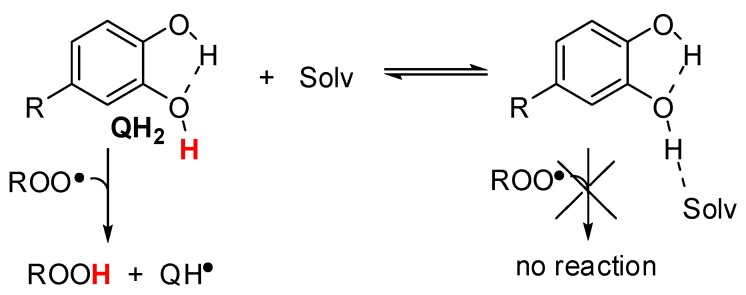
Kinetic solvent effect explaining the reduced reactivity in H-bond accepting solvents (Solv).

Based on this mechanism, the decrease in reactivity recorded on moving from PhCl to MeCN to buffered water as the reaction medium, is perfectly explained by the progressively stronger hydrogen-bonding of the “reactive” OH group to the solvent (Scheme 2), thereby causing a decrease in its reactivity [26,34,35]. 

Interestingly, when the pH was raised to 7.4, the reactivity of quercetin increased significantly. Oxygen-uptake plots showed a neat inhibition period corresponding to the trapping of four peroxyl radicals. The first half of the inhibition period provided a *k*_inh_ of 1.6 × 10^5^ M^−1^ s^−1^ (i.e., 40-fold higher than at pH 2.1) matching the reactivity of reference PMHC. The analysis of the second half of the inhibition period afforded a somewhat reduced *k*_inh_ of 7 × 10^4^ M^−1^ s^−1^, yet about 20-fold faster than at pH 2.1. Such a major boost in the antioxidant performance clearly suggests a change in the reaction mechanism, and can be compared to the negligible change in reactivity of monophenolic PMHC and to the modest enhancement of reactivity for catechol, whose *k*_inh_ grows only by a factor of 2 (Table 1) on raising the pH from 2.1 to 7.4 [26]. Indeed, at pH 7.4, the reactivity of quercetin surpasses that of catechol by over one order of magnitude and the stoichiometric factor is approximately doubled. 

Despite its modest magnitude, the enhanced reactivity of catechol upon raising the pH was explained by partial deprotonation which yielded the more electron-rich phenoxide anion [26]; somewhat similarly, the enhanced reactivity of quercetin with DPPH^•^ radical in ionizing solvents, as compared with non-ionizing solvents of similar H-bond accepting ability, was explained by deprotonation of the OH group in position 7 which yielded an electron-rich phenoxide that would undergo fast ET to the oxidizing radical (Scheme 1, Path B) [25,36]. However, in quercetin, this points toward the major role of the OH in 7 rather than the catechol. On the other hand, previous studies underline the importance of the OH in position 3: the reaction of quercetin toward the radical of a synthetic analogue of α-tocopherol in ethanol was found to be 29-fold faster than that of rutin, where the OH in 3 is glycosylated and unavailable for reaction [37]. To shed some light on these mechanistic possibilities and identify the most stable anions and transient intermediates of quercetin, we turned to quanto-mechanical calculations.

### 3.2. Quanto-Mechanical Calculations on Quercetin

To help identify the most likely mechanism for reaction of quercetin with peroxyl radicals in water, we first calculated the most stable transient intermediates of quercetin and its main anions in the gas phase and in water solution, based on the relative free energies of formation, by using a PCM, at B3LYP/6-311+g (d,p) level, which had previously been shown to be reliable for phenolic compounds [29,30]. Subsequently, we referred to the time-resolved UV–vis spectra obtained by pulse radiolysis by Jovanovic and co-workers during the reaction of quercetin with N_3_^•^ radical in water at various pH [38]. In order to assign the experimental spectra to specific phenoxyl radicals of quercetin, we matched them with the spectra that we calculated for any transient, using TD-DFT methods, which are emerging as powerful tools to investigate radical reactions by allowing the assignment of transient UV–vis spectra [39,40,41,42].

#### 3.2.1. Neutral Radicals from Quercetin

**Figure 2:**
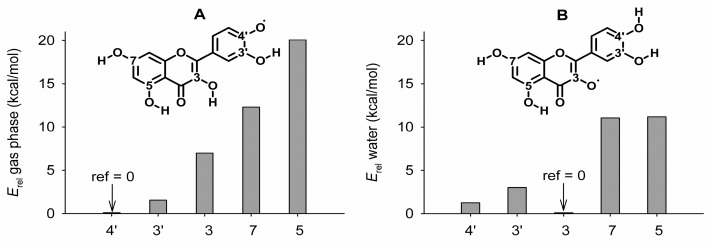
Relative stability of the neutral radicals obtained by H-atom abstraction from quercetin, calculated in the gas phase (**A**) and in water (**B**), determined from the relative energy (*E*_rel_) with reference to the most stable species (ref = 0). Numbers on the *x*-axis indicate the position of the radical.

Upon removing a H-atom from a quercetin’s OH group, five neutral phenoxyl radicals can be formed, whose relative stabilities are reported in Figure 2 (see Figure S1 for all the structures). 

In the gas phase, the most stable radical is in position 4’, because the –O^•^ moiety can accept a relatively strong intramolecular H-bond from the neighboring –OH group and the spin density can be delocalized in ring C (see Figure S2). Calculations performed by using water as the implicit solvent showed that the radical in position 3 is more stable than that in position 4′ (+1.2 kcal/mol), while the other three radicals (in positions 3’, 7 and 5) have higher energies (by +3.0, +11.0, +11.2 kcal/mol, respectively), in line with previous reports [43]. The inversion of the stabilities of radicals in positions 4′ and 3, on moving from the gas phase to water, is due to the high polarity of the catechol (ring B), which is more strongly solvated than the –OH in position 3, which donates an intramolecular H-bond to the neighboring carbonyl. In general, compared to a “free” OH, the cleavage of an intramolecular H-bonded –OH is more energetically costly in the gas phase than in water.

**Figure 3:**
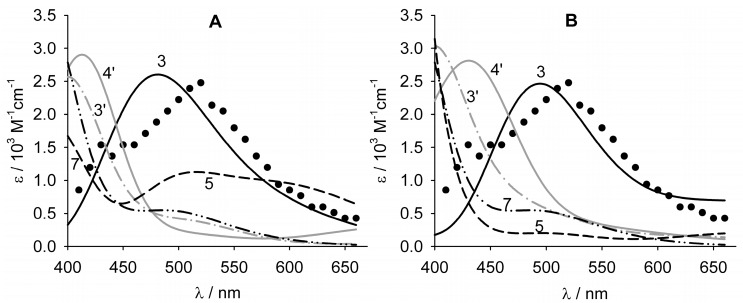
Calculated ultraviolet–visible (UV–vis) spectra of the neutral radicals of quercetin in the gas phase (**A**) or in water (**B**), compared to the experimental spectrum measured at pH 2.6 (●) by Jovanovich et al. [38]. Numbers indicate the position of the radical.

The UV–vis spectra of the radicals of quercetin were measured by pulse radiolysis by Jovanovic et al. [38], by reacting quercetin with the N_3_^•^ radical in water at various pH values. The changes of the transient spectra in the pH range 2.6 < pH < 12 showed that the phenoxyl radical from quercetin has two deprotonation equilibria, at p*K*_a_ = 4.2 and 9.4, which were attributed, respectively, to the equilibria between the neutral phenoxyl radical and the radical anion and that between the radical anion and the corresponding radical dianion [38]. The spectrum recorded at pH 2.6 is characterized by a λ_max_ of 515 nm and a shoulder at 440 nm (see Figure 3). To assign the structure of the radical originating from the spectrum recorded at pH 2.6, the UV–vis absorption spectra of all neutral radicals which could be formed after H-atom abstraction from quercetin were calculated, and the matching with the experimental spectrum is shown in Figure 3.

Both the calculations in gas phase and water agree, indicating that the best fit of the experimental spectrum at pH = 2.6 is given by the radical formed in position 3. It should be noted that the quality of the matching of simulated spectra improves in water, since calculations predict a bathochromic shift due to dipolar interaction with the solvent (particularly for radicals in positions 3 and 4′). In water, the radical in position 3 has two strong transitions at 500 and 477 nm, that originate an absorbance peak at 495 nm, that is very near to the maximum of 515 nm determined by pulse radiolysis (Figure 3B). Interestingly, this assignment agrees with the radical stabilities reported in Figure 2. In Figure 3, it is also evident that the radicals in positions 5, 7 and 3′ have the most significant transitions below 400 nm, so their spectra do not reproduce the shape of the experimental spectra. Many studies have assumed that, upon H-atom abstraction from quercetin, the most stable radical formed is that in position 4’. As a matter of fact, in water, this radical has two strong transitions at 442 and 396 nm, which create an absorption band at 430 nm, far from the experimental maximum. Nonetheless, small amounts of the radical in position 4’, which is calculated to be only 1.2 kcal/mol less stable than that of the radical in position 3, may be present in solution, and it likely originates from the shoulder at 440 nm visible in the experimental spectrum. Assuming that the shoulder at 440 nm can be entirely attributed to the radical in position 4′, from the relative areas of the two bands at 515 and 440 nm, it can be estimated that the ratio of the two radicals in positions 3 and 4′ is acidic solution is approximately 9:1.

#### 3.2.2. Radical Anions from Quercetin

**Figure 4:**
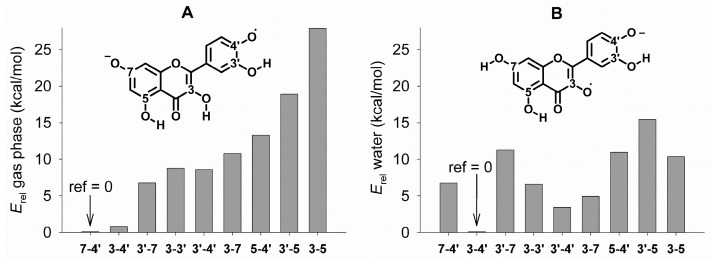
Relative stability of the radical anions obtained by H-atom abstraction from deprotonated quercetin, calculated in the gas phase (**A**) and in water (**B**), determined from the relative energy (*E*_rel_) with reference to the most stable species (ref = 0). Numbers on the *x*-axis indicate the position of the radical and of the negative charge. The radical anion in 5-7 is omitted for clarity (*E*_rel_ is 32.7 and 21.0 kcal/mol in the gas phase and water, respectively).

The stabilities of all the possible ten radical anions were calculated and the results are reported in Figure 4 (see Figure S3 for the structures).

In the gas phase, the most stable tautomer is the radical anion in position 7-4’, while the radical anion in position 3-4′ is less stable by 0.8 kcal/mol. In water, the stability order is reversed, as the radical anion in position 3-4’ is more stable than that in position 7-4′ by 6.8 kcal/mol. Similar to what was said in the case of the neutral radicals, upon moving from gas phase to water, the loss of a H-atom from the “free” 7–OH is made more energetically expensive, while that from the 3–OH, that is protected by an intra-molecular H-bond, becomes relatively easier. Interestingly, the radical anion involving the catechol on ring B (i.e., in position 3-4’) that has been proposed by Jovanovic as a putative structure for the quercetin radical at pH 5.3 [38], is predicted to be less stable than the radical anion in position 7-4’ by 8.6 kcal/mol in the gas phase, while in water it is less stable than the radical anion in position 3-4’ by 3.4 kcal/mol. 

**Figure 5:**
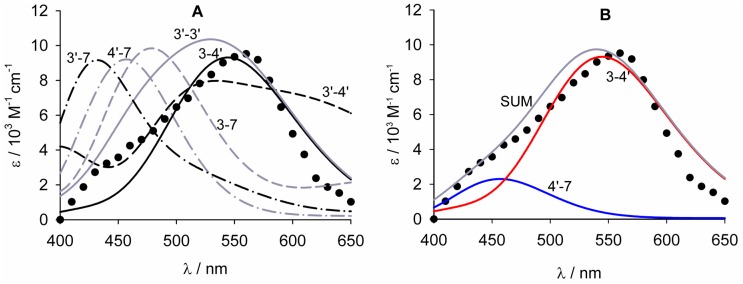
(**A**) Calculated UV–vis spectra of the radical anions of quercetin in water, compared to the experimental spectrum measured at pH 5.8 (●) by Jovanovich et al. [38]. The spectra for the tautomers involving the 5–OH are omitted for clarity. (**B**) Simulation of the spectrum arising from the overlay of radical anions in positions 3-4′ and 4′-7 in relative ratio 3.2:1 (SUM). Numbers indicate the position of the radical and of the negative charge.

The experimental UV–vis spectrum of quercetin, recorded by pulse radiolysis at pH 5.8, shows an absorption maximum at 557 nm and a shoulder at about 450 nm. In Figure 5, this spectrum is compared to those calculated for the most stable radical anions.

Considering the spectra calculated in water, which seems the most appropriate approach in the case of charged species, the radical anion that more closely matches the experimental spectrum is that involving the positions 3-4′. Its calculated λ_max_ at 547 nm originates from two transitions at 538 and 561 nm. The assignment of the experimental spectrum to the radical anion in position 3-4′ agrees with the relative stabilities of the various species reported in Figure 4. The λ_max_ for the radical anion in position 3-3′ (see Figure 5) is at lower wavelength (530 nm) than that in position 3-4′, due to the convolution of the main transition at 554 nm with minor transitions between 470 and 512 nm. This radical anion was calculated to be less stable than that in position 3-4′ by 6.6 kcal/mol. The radical anion in position 3-4′ has two transitions, with comparable intensity at 513 and 620 nm, originating from a large absorption band which does not reproduce the shape of the experimental spectrum. This radical is less stable than that in position 3-4′ by 3.4 kcal/mol. The radical anions involving the 5–OH (not shown in Figure 5) are destabilized by at least 10 kcal/mol with respect to the radical anion in position 3-4′, and have the main absorption peaks at about 470 nm. All radical anions involving the 7–OH group show absorption maxima between 435 and 480 nm, that may be responsible for the shoulder at 450 nm visible in the experimental spectrum. Considering the radical anion in position 4′-7 as the one showing the closest electronic transition, we obtained a good matching of the experimental spectrum by simulating the UV–vis spectrum that would arise from the mixture of radical anions in positions 3-4′ and 4′-7 in the ration of approximately 3:1 (Figure 5B).

### 3.3. Bond Dissociation Enthalpies 

To rationalize the reactivity toward peroxyl radicals, the dissociation enthalpy of the OH bonds of quercetin and of the quercetin anion was computed in water. Undissociated quercetin reacts preferentially at the 3–OH group, with a BDE_OH_ of 83.1 kcal/mol (Table 2).

Concerning the BDE_OH_ of dissociated quercetin, calculations indicated that the most acidic –OH group is that in position 4’, followed by those in positions 7 (+1.6 kcal/mol), 3′ (3.2 kcal/mol), 3 (+3.9 kcal/mol) and 5 (+6.9 kcal/mol), in agreement with previous calculations [44]. The BDE of all –OH groups of the two most stable anions were then calculated as reported in Table 2. The results indicate that both anions in positions 4′ and 7 preferentially react in position 3, its BDE_OH_ being 75.0 and 78.7 kcal/mol, respectively. The results in Table 2 justify that deprotonation boosts the reactivity of quercetin by lowering the BDE_OH_.

### 3.4. A Proposed Mechanism for the Antioxidant Activity of Quercetin in Water

The reactivity of quercetin in acidic medium is superimposable to that of simple catechol, hence it most likely involves the catechol moiety. Calculations indicated that the most stable transient is the radical in position 3, which is more stable than the radical in position 4′ by as little as 1.2 kcal/mol: a difference of marginal relevance considering the difficulty to reproduce the aqueous medium with a PCM. 

**Scheme 3:**
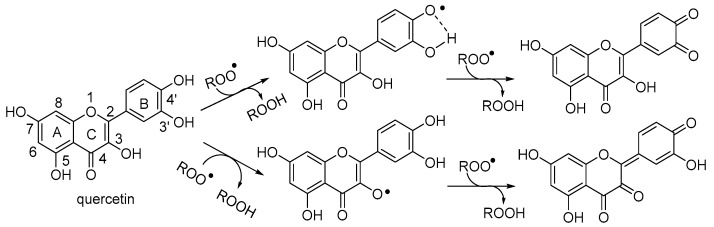
Mechanistic proposal for the observed antioxidant activity of quercetin in water at pH = 2.1.

Considering that both the radicals in positions 3 and 4′ coexist in the transient UV spectrum obtained by pulse radiolysis and that the modest difference in BDE_OH_ (0.2 kcal/mol, see Table 2) is unlikely to be visible in kinetic measurements, our data indicate that both reaction pathways summarized in Scheme 3 could coexist and contribute to the observed reactivity.

**Scheme 4:**
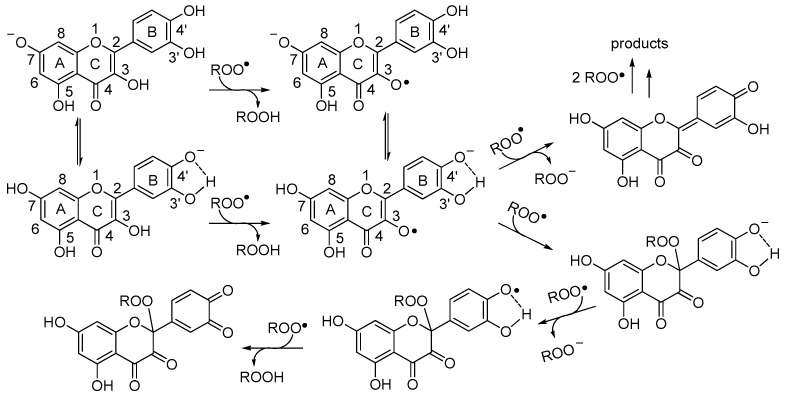
Mechanistic proposal for the observed antioxidant activity of quercetin in water at pH = 7.4.

At close to neutral pH, instead, the reactivity of quercetin is far less simple than Scheme 1 would suggest. The trapping of four peroxyl radicals with different rate constants indicates that two distinct moieties are independently reacting with two radicals each: namely, the chromen-4-one core (rings A + C) and the catechol moiety (ring B). Clearly, deprotonation has a major role and a rate-determining reaction with peroxyl radicals must occur from the mono-anion, as was previously recognized [25]. Litwinienko and coworkers reported that the p*K*_a_ of quercetin in water/methanol is 8.45, indicating that a significant portion would be dissociated at pH 7.4, and identified position 7 as the most acidic [25]. Our calculations, as well as others [44], suggest instead that the anion in position 4′ would be marginally more stable (again, some approximation in the calculations’ model is to be considered). Interestingly, both the anions in positions 4′ and 7 have the lowest BDE_OH_ in position 3, which matches the assignment of pulse radiolysis transients as a mixture of radical anions in positions 4′-3 and 7-3 in different ratios (main signal and shoulder, respectively). On this basis, we suggest that both equilibrating anions contribute to the observed reactivity as tentatively illustrated in Scheme 4. For each route and for the trapping of each of the four peroxyl radicals, current data do not allow to clearly distinguish whether the reaction occurs by concerted EPT or stepwise PT/ET, and most likely a combination of the two mechanisms is operating.

## 4. Conclusions

The antioxidant activity of quercetin in water strongly depends on pH, which affects both its absolute performance and its mechanism. Under acidic conditions (pH = 2.1), the antioxidant performance is modest and comparable to simple catechol: its mechanism appears to involve the trapping of two peroxyl radicals by the catechol moiety or by the catechol moiety (in position 4′) and by the –OH in position 3, which are the most reactive sites. Under more biomimetic settings (pH = 7.4), however, the antioxidant performance is boosted 40-fold, approaching that of α-tocopherol mimic PMHC and trapping twice as many (i.e., four) peroxyl radicals: two in the chromen-4-one core and two in the catechol moiety. The fastest rate-controlling reaction with peroxyl radicals comes from both equilibrating mono-anions in position 4′ (in the catechol) and position 7 (in the chromen-4-one core) and involves the –OH in position 3. Although the mono-anion may account for only about 10% of the quercetin in solution at pH 7.4, based on the reported p*K*_a_ of 8.45 [25] (lower p*K*_a_ values have also been reported [45]), the much higher reactivity of such an electron-rich structure would overwhelm that of the undissociated form, driving the whole process and progressively shifting the deprotonation equilibrium. Formal H-atom transfer to peroxyl radicals is likely to occur by a combination of concerted EPT and of stepwise PT/ET (i.e., SPLET) mechanisms, both being favored under such settings. The obtained results help explain and support the relevance of quercetin as a nutritional antioxidant. They will also prove useful in the rational design of novel catechol-based bioinspired antioxidants. 

## Figures and Tables

**Table 1:** Rate constants *k*_inh_ and stoichiometric factors *n* for the trapping of peroxyl radicals by quercetin and reference antioxidants, measured in the inhibited autoxidation of tetrahydrofuran (THF) in buffered water at 303 K. Reference values in other solvents are reported for comparison.

	Water				MeCN		PhCl	
	pH = 2.1		pH = 7.4					
	*k*_inh_ (M^−1^ s^−1^)	*n*	*k*_inh_ (M^−1^ s^−1^)	*n*	*k*_inh_ (M^−1^ s^−1^)	*n*	*k*_inh_ (M^−1^ s^−1^)	*n*
Quercetin	(4.0 ± 0.5) × 10^3^	-	(1.6 ± 0.3) × 10^5^	2.1 ± 0.2	1.2 × 10^4 a^	2.1	5.6 × 10^5 b^	2.1
			(7.0 ± 0.5) × 10^4^	2.0 ± 0.2				
Catechol ^c^	3.0 × 10^3^	-	7.0 × 10^3^	-	2.5 × 10^4^	2	5.5 × 10^5 b^	2.0
PMHC ^c^	1.9 × 10^5^	1.8	2.0 × 10^5^	1.8	6.8 × 10^5^	2	3.2 × 10^6^	2.0

^a^ Data from reference [22]. ^b^ Data from reference [23]. ^c^ Data from reference [26]. PMHC: 2,2,5,7,8-Pentamethyl-6-chromanol.

**Table 2:** Bond dissociation enthalpies (BDE_OH_) of quercetin and of its anions, calculated in water at B3LYP/6-311+g (d,p) level using the isodesmic approach.

Starting Compound	Abstracted OH	BDE_OH_ (kcal/mol)
Neutral Quercetin	4′	83.3
	3′	86.0
	3	83.1
	7	94.0
	5	94.7
Anion 4′	7	81.2
	3′	79.1
	3	75.0
	5	86.4
Anion 7	4′	79.5
	3′	84.8
	3	78.7
	5	95.7

## Supplementary Materials

The following are available online at www.mdpi.com/2313-7673/2/3/9/s1, Figure S1: Structures of the anions and of the neutral phenoxyl radicals of quercetin, Figure S2: Spin distribution of quercetin radicals, Figure S3: Structures of the radical anions of quercetin, Appendix A: Details on quanto-mechanical calculations.
