# Supplementary material for: The Antioxidant Activity of Quercetin in Water Solution
Source: Biomimetics (Basel). 2017 Jun 27;2(3):9. doi: 10.3390/biomimetics2030009 (PMC6352608; doi:10.3390/biomimetics2030009)
Supplement: Supplementary file 1 [file biomimetics-02-00009-s001.pdf]

Supplementary Materials: The Antioxidant Activity of Quercetin in Water Solution

Riccardo Amorati [1,*], Andrea Baschieri [1], Adam Cowden [2] and Luca Valgimigli [1,*]

[1] University of Bologna, Department of Chemistry "G. Ciamician", Via S. Giacomo 11, 40126 Bologna, Italy; andrea.baschieri2@unibo.it

[2] School of Chemistry (Rm 267), University of Edinburgh, West Mains Road, Edinburgh EH9 3FJ, UK; adamcowden@gmail.com

* Correspondence: riccardo.amorati@unibo.it (R.A.); luca.valgimigli@unibo.it (L.V.); Tel.: +39-051-209-5689 (R.A.); +39-051-209-5683 (L.V.)

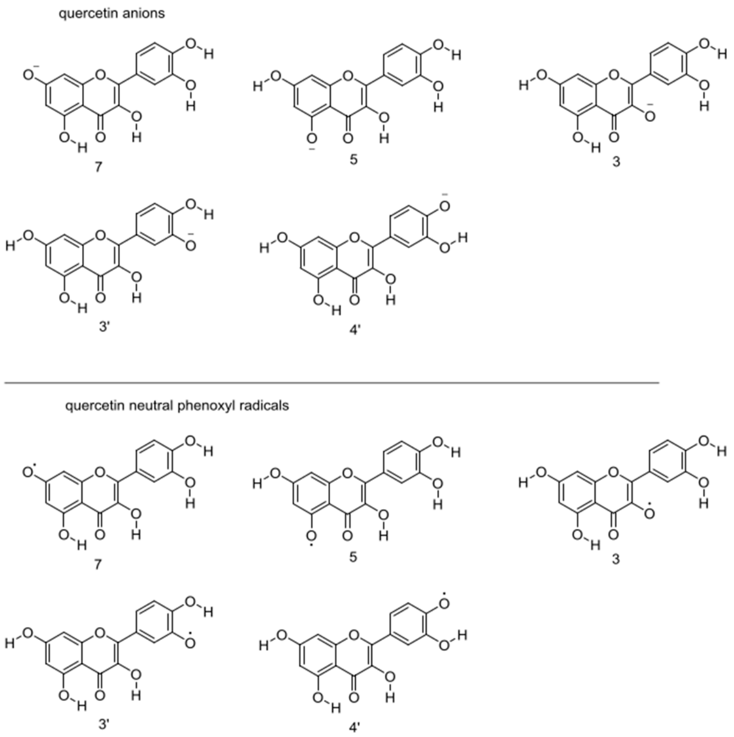

Figure S1. Structures of the anions and of the neutral phenoxyl radicals of quercetin.

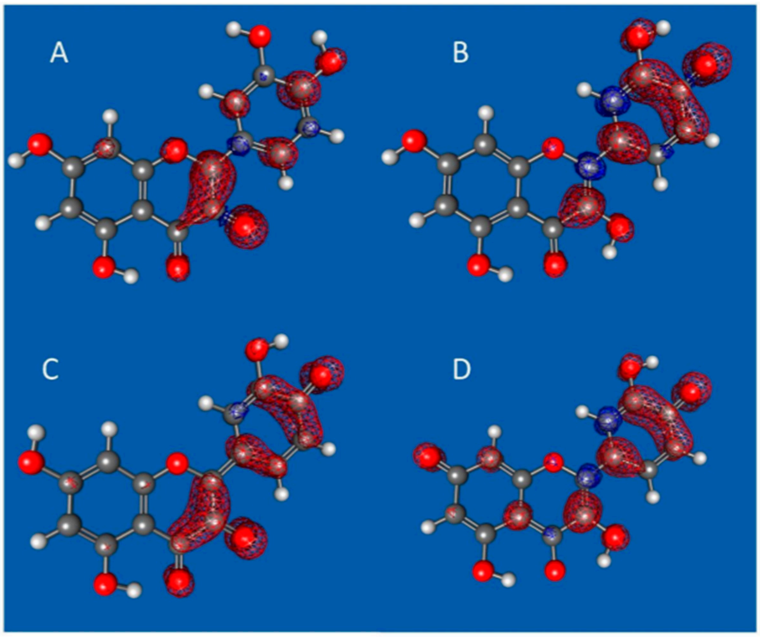

Figure S2. Spin distribution of quercetin radicals. Neutral radicals (**A**) 3–OH and (**B**) 4′–OH, and radical anions (**C**) 3,4′ and (**D**) 7,4′ of quercetin.

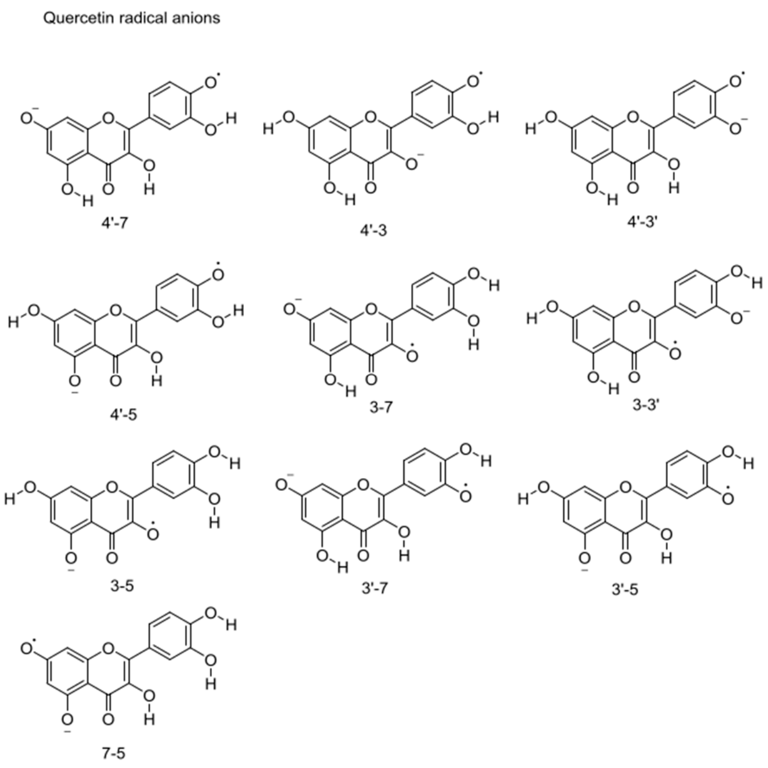

Figure S3. Structures of the radical anions of quercetin.

Appendix A. Details on quanto-mechanical calculations

Cartesian atomic coordinates, thermal correction to Enthalpy, thermal correction to Gibbs Free Energy, single point electronic energies in the gas phase, total free energy in solution with all non-electrostatic terms, excitation energies, and oscillator strengths (all energies are in Hartrees).

Appendix A1. Quercetin

B3LYP/6-31+g(d,p) gas phase

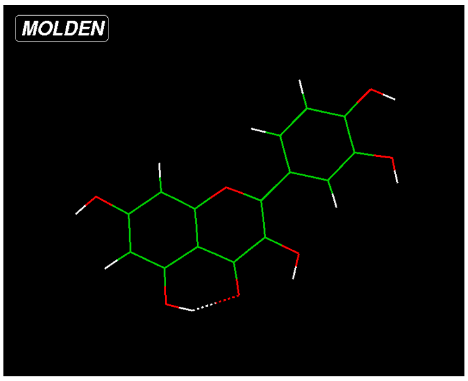

Coordinates:
C,0,-0.0078071959,-0.2910001478,0.0354204811\C ,0,0.0063972896,-0.1101873431,1.4145395653\C,0,1.2030198006,0.0584779642,2.137511677\C,0,2.4376714705,0.0426733324,1.4284629163\C,0,2.441209 877,-0.1370469386,0.0492697546\C,0,1.2215243701,-0.3013335742,-0.62916 0678\O,0,-1.1931496794,-0.1008115556,2.0576005531\ C,0,-1.3105990374,0.0692129039,3.4167369365\ C,0,-0.1733487801,0.2375716181,4.1656273012\ C,0,1.1424756376,0.2401385033,3.5587304268\C,0,-2.7042012003,0.040993469 7,3.8640567551\C,0,-3.0437489202,0.197720567,5.2269802045\C,0,-4.3718383627,0.1668026031,5.6224199363\C,0,-5.4032197343,-0.0194259845,4.6861 938121\C,0,-5.0754268878,-0.1747024843,3.339981163\C,0,-3.7450301314,- 0.1453112871,2.9317396581\O,0,-6.7047624162,-0.049130089,5.0783051859\ O,0,-4.7921091481,0.3109369843,6.9272628041\O,0,-0.1798179732,0.413225 3216,5.5150626314\ O,0,2.154612756,0.3996030163,4.2990336765\O,0,3.5984 954905,0.2005342064,2.0856698183\ O,0,1.1798805265,-0.4800373784,-1.979 0484292\H,0,-0.9351572163,-0.4195183347,-0.508055014\H,0,3.3886270904, -0.147364379,-0.4809468675\ H,0,0.766864687,0.5031308769,5.753382007\ H,0,3.3859573833,0.3127867801,3.0472672791\ H,0,2.0743226,-0.4722815034,- 2.345509958\H,0,-3.5110738982,-0.2676976015,1.8823005101\H,0,-5.876333 0998,-0.3180964542,2.6223148804\H,0,-2.267990637,0.3426561172,5.968440 0827\H,0,-6.7439808571,0.0738401339,6.0395107583\H,0,-4.0375103265,0.4347725366,7.5171893773

Thermal correction to Enthalpy= 0.244747
Thermal correction to Gibbs Free Energy= 0.179105
Lowest Frequency: 19.3458 cm-1

B3LYP TD(nstates=10) /6-311+g(d,p)
HF=-1104.5156848

Excitation energies and oscillator strengths:

Excited State	1:	Singlet-A	3.2984 eV	375.89 nm	f=0.4668
Excited State	2:	Singlet-A	3.7065 eV	334.50 nm	f=0.0875
Excited State	3:	Singlet-A	4.1529 eV	298.55 nm	f=0.0569
Excited State	4:	Singlet-A	4.2495 eV	291.76 nm	f=0.0145
Excited State	5:	Singlet-A	4.4283 eV	279.98 nm	f=0.0000
Excited State	6:	Singlet-A	4.5696 eV	271.32 nm	f=0.0721
Excited State	7:	Singlet-A	4.7582 eV	260.57 nm	f=0.0010
Excited State	8:	Singlet-A	4.7810 eV	259.33 nm	f=0.0859
Excited State	9:	Singlet-A	4.8587 eV	255.18 nm	f=0.2427
Excited State	10:	Singlet-A	4.9676 eV	249.58 nm	f=0.0514

B3LYP TD(nstates=10) /6-311+g(d,p) scrf=(pcm,solvent=water)

Total free energy in solution: with all non-electrostatic terms (a.u.) = -1104.538472

Excited State	1:	Singlet-A	3.1891 eV	388.78 nm	f=0.5963
Excited State	2:	Singlet-A	3.6238 eV	342.14 nm	f=0.0443
Excited State	3:	Singlet-A	4.0581 eV	305.52 nm	f=0.1013
Excited State	4:	Singlet-A	4.2127 eV	294.31 nm	f=0.0334
Excited State	5:	Singlet-A	4.5203 eV	274.29 nm	f=0.0000
Excited State	6:	Singlet-A	4.5319 eV	273.58 nm	f=0.1161
Excited State	7:	Singlet-A	4.7547 eV	260.76 nm	f=0.1081
Excited State	8:	Singlet-A	4.8235 eV	257.04 nm	f=0.2781
Excited State	9:	Singlet-A	4.9521 eV	250.37 nm	f=0.0648
Excited State	10:	Singlet-A	5.0066 eV	247.64 nm	f=0.0561

Appendix A2. Quercetin anion 3–OH

B3LYP/6-31+g(d,p) gas phase

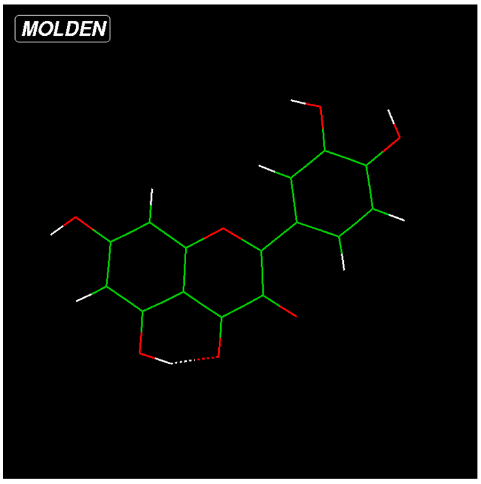

Coordinates:

C,0,0.0131867979,0.0000236305,-0.0027265828\ C,0,0.010961563,0.0000635511,1.4031508721\ C,0,1.2026364,0.0001749924,2.1513754718\C,0,2.4441800041,0.0000581137,1.4429794258\C,0,2.460183 4784,0.0000603272,0.0492112974\C,0,1.2403140518,0.0000566433,-0.6515908374\O,0,-1.175282915,0.0000522071,2.0230766642\C,0,-1.2930821963,- 0.0000070669,3.4203708848\C,0,-0.162603546,-0.0000114215,4.2811745853\ C,0,1.1714608723,0.0005603602,3.5995087251\C,0,-2.6863314895,0.0000592124,3.8263481115\C,0,-3.7232232363,0.0000994583,2.8576392\C,0,-5.0570592603,0.0001336243,3.2399875534\C,0,-5.4284402633,0.0001394423,4.5884326722\C,0,-4.4223297703,0.0001010799,5.5529967748\C,0,-3.0753437983,0.0000612135,5.1908939955\O,0,-6.759949381,0.0001746694,4.9565133319\O,0,-6.1107645389,0.0001672702,2.3267990346\O,0,-0.2203459536,0.000459893,5.5434407633\O,0,2.2488069637,0.0003227239,4.2581479034\O,0,3.5939027 972,-0.0001917672,2.1350504829\O,0,1.2255526147,0.0000177665,-2.0319066158\H,0,-0.9156524318,-0.0000144712,-0.5601278375\H,0,3.4139972465,-0.0000013919,-0.4719993364\H,0,3.302004331,-0.0006360679,3.1156235194\H,0,2.1372504459,0.0000191068,-2.3498394684\H,0,-2.2921896271,0.0000214799,5.9383421861\H,0,-4.7086397312,0.0000993899,6.6011673253\H,0,-3.4721748378,0.0000952527,1.8010933778\H,0,-5.7494848782,0.0001606306,1.4319653031\H,0,-7.2848663356,0.0001947088,4.1431852818

Thermal correction to Enthalpy= 0.229758
Thermal correction to Gibbs Free Energy= 0.164168
Lowest freq: 32.0787 cm-1

B3LYP TD(nstates=12) /6-311+g(d,p)
HF=-1103.969728

Excited State	1:	Singlet-A	2.5533 eV	485.58 nm	f=0.3648
Excited State	2:	Singlet-A	2.6696 eV	464.43 nm	f=0.0003
Excited State	3:	Singlet-A	2.8624 eV	433.15 nm	f=0.0000

Excited State	4:	Singlet-A	2.9316 eV	422.92 nm	f=0.0001
Excited State	5:	Singlet-A	3.2691 eV	379.26 nm	f=0.0108
Excited State	6:	Singlet-A	3.5032 eV	353.92 nm	f=0.0001
Excited State	7:	Singlet-A	3.5235 eV	351.87 nm	f=0.0726
Excited State	8:	Singlet-A	3.8672 eV	320.60 nm	f=0.0442
Excited State	9:	Singlet-A	3.8778 eV	319.73 nm	f=0.0000
Excited State	10:	Singlet-A	3.9512 eV	313.79 nm	f=0.0003
Excited State	11:	Singlet-A	4.0596 eV	305.41 nm	f=0.0000
Excited State	12:	Singlet-A	4.0715 eV	304.51 nm	f=0.0020

B3LYP TD(nstates=12) /6-311+g(d,p) scrf=(pcm,solvent=wat er)

Total free energy in solution: with all non-electrostatic terms (a.u.) = -1104.060891

Excited State	1:	Singlet-A	2.6905 eV	460.83 nm	f=0.5259
Excited State	2:	Singlet-A	3.4480 eV	359.59 nm	f=0.0001
Excited State	3:	Singlet-A	3.7648 eV	329.32 nm	f=0.0016
Excited State	4:	Singlet-A	3.8019 eV	326.12 nm	f=0.0343
Excited State	5:	Singlet-A	3.9544 eV	313.53 nm	f=0.0716
Excited State	6:	Singlet-A	4.0894 eV	303.19 nm	f=0.0003
Excited State	7:	Singlet-A	4.1462 eV	299.03 nm	f=0.0143
Excited State	8:	Singlet-A	4.2248 eV	293.47 nm	f=0.1529
Excited State	9:	Singlet-A	4.3166 eV	287.23 nm	f=0.0000
Excited State	10:	Singlet-A	4.4461 eV	278.86 nm	f=0.1150
Excited State	11:	Singlet-A	4.5422 eV	272.96 nm	f=0.0000
Excited State	12:	Singlet-A	4.5843 eV	270.45 nm	f=0.0024

Appendix A3. Quercetin anion 3′–OH

B3LYP/6-31+g(d,p) gas phase

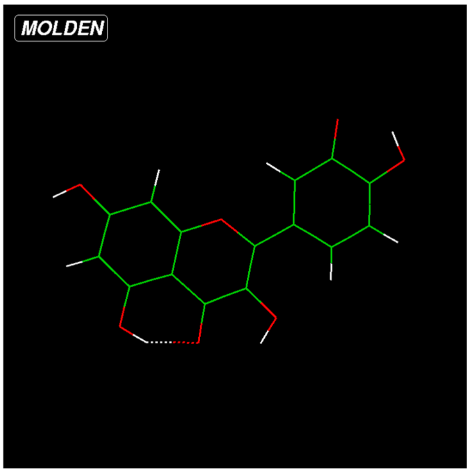

Coordinates:
C,0,-0.0206146779,0.0822931545,0.0113391735\ C,0,-0.0121614738,0.0303429173,1.4080353232\ C,0,1.1814629241,-0.0224817552,2.1436394868\ C,0,2.4181149159,-0.0231353673,1.4474470991\C,0,2.4299380824,0.0281869349,0.0553137672\C,0,1.2102427036,0.0801241495,-0.6415225135\C,0,1.1108449736,-0.0743085248,3.5809788705\C,0,-0.1851608055,-0.0686467127,4.1757959699\C,0,-1.3506396393,-0.0155385328,3.4171573702\O,0,-1.2077362758,0.0323762583,2.0431934939\C,0,-2.7368999414,-0.0013406381,3.8342703831\C,0,-3.0712218159,-0.0451652256,5.2114922483\C,0,-4.4148126328,-0.0308100027,5.6032997769\C,0,-5.4114180745,0.026189291,4.6394944024\C,0,-5.1312099455,0.0728198467,3.2156818644\C,0,-3.7703087577,0.057082116,2.8549683899\O,0,-6.1622615412,0.1242405763,2.441879986\O,0,-6.732972036,0.0439918427,4.9208736876\O,0,2.1550610524,-0.1243108899,4.3213728124\O,0,-0.1863309303,-0.1193791534,5.5432491594\O,0,3.5752523683,-0.0734021323,2.137623278\O,0,1.1864960017,0.1315108566,-2.0143969915\H,0,-0.9511153555,0.1226780955,-0.5408270467\H,0,3.381392325,0.0272788285,-0.4694440548\H,0,0.7645794967,-0.1482383634,5.7729372311\H,0,3.3187912628,-0.1036271566,3.1056752771\H,0,2.0926595307,0.1244788684,-2.3482465767\H,0,-2.2964310704,-0.0895474384,5.9619780037\H,0,-4.6835589279,-0.0640295356,6.6562901995\H,0,-3.5253775223,0.091008246,1.8006887895\H,0,-7.1017649577,0.0859459237,3.9901684622

Thermal correction to Enthalpy= 0.230739

Thermal correction to Gibbs Free Energy= 0.164478

Lowest freq: 3.8592 cm-1

B3LYP TD(nstates=12) /6-311+g(d,p)

HF=-1103.985417

Excitation energies and oscillator strengths:

Excited State	1:	Singlet-A	1.8633 eV	665.39 nm	f=0.1248
Excited State	2:	Singlet-A	2.7082 eV	457.82 nm	f=0.0000

Excited State	3:	Singlet-A	2.7317 eV	453.87 nm	f=0.0002
Excited State	4:	Singlet-A	3.0308 eV	409.07 nm	f=0.0000
Excited State	5:	Singlet-A	3.1312 eV	395.96 nm	f=0.4551
Excited State	6:	Singlet-A	3.2826 eV	377.70 nm	f=0.1053
Excited State	7:	Singlet-A	3.4707 eV	357.23 nm	f=0.0006
Excited State	8:	Singlet-A	3.8840 eV	319.22 nm	f=0.0001
Excited State	9:	Singlet-A	3.9049 eV	317.51 nm	f=0.0018
Excited State	10:	Singlet-A	3.9293 eV	315.54 nm	f=0.0014
Excited State	11:	Singlet-A	3.9687 eV	312.40 nm	f=0.0000
Excited State	12:	Singlet-A	4.0352 eV	307.26 nm	f=0.0413

B3LYP TD(nstates=12) /6-311+g(d,p) scrf=(pcm,solvent=wat er)

Total free energy in solution: with all non-electrostatic terms (a.u.) = -1104.062933

Excited State	1:	Singlet-A	2.4962 eV	496.70 nm	f=0.3070
Excited State	3:	Singlet-A	3.7005 eV	335.05 nm	f=0.0905
Excited State	4:	Singlet-A	3.7450 eV	331.06 nm	f=0.0003
Excited State	5:	Singlet-A	3.9940 eV	310.43 nm	f=0.0000
Excited State	6:	Singlet-A	4.1959 eV	295.49 nm	f=0.0498
Excited State	7:	Singlet-A	4.2936 eV	288.77 nm	f=0.0464
Excited State	8:	Singlet-A	4.3045 eV	288.03 nm	f=0.0009
Excited State	9:	Singlet-A	4.5282 eV	273.81 nm	f=0.0000
Excited State	10:	Singlet-A	4.5471 eV	272.66 nm	f=0.0567
Excited State	11:	Singlet-A	4.5743 eV	271.05 nm	f=0.0000
Excited State	12:	Singlet-A	4.6961 eV	264.01 nm	f=0.1417

Appendix A4. Quercetin anion 4′–OH

B3LYP/6-31+g(d,p) gas phase

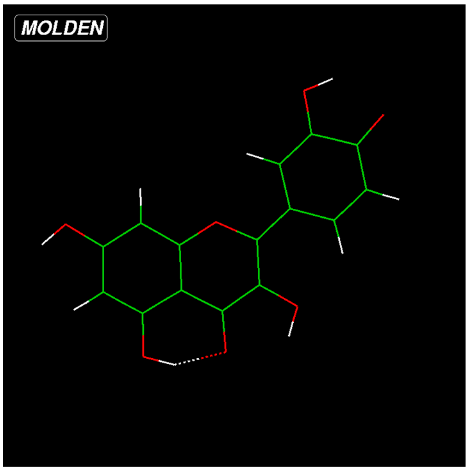

Coordinates:
C,0,0.0153326944,-0.0008669633,-0.0203593888\C,0,0.0070812045,-0.0003153334,1.3773023165\C,0,1.1909509824,0.00027001,2.1279141938\C,0,2.4343655938,0.0002908698,1.4475849615\C,0,2.4642323331,-0.0002415826,0.0526008216\C,0,1.2543782389,-0.0008128757, -0.6594374299\C,0,1.1058808781,0.0008536841,3.5698856883\C,0,-0.187096276,0.0007855866,4.1471026205\C,0,-1.355256542,0.0001803988,3.3771552454\O,0,-1.1994334405,-0.0003502669,1.9978936059\C,0,-2.7249623935,0.0000185596,3.7828410366\C,0,-3.1087339273,0.0005038115,5.1559787405\C,0,-4.4374306083,0.0003439876,5.539366615\C,0,-5.4994749576,-0.0003234332,4.580002121\C,0,-5.0830766207,-0.0008020402,3.1830747833\C,0,-3.770428871,-0.0006449702,2.7975320645\O,0,-6.1200359853,-0.0014279226,2.2929621773\O,0,-6.7470168445,-0.0004874115,4.8288941261\O,0,2.1519602766,0.0014987772,4.3176702533\O,0,-0.213170659,0.0013549255,5.5193123546\O,0,3.5829898656,0.0008026916,2.1539646226\O,0,1.2449379475,-0.0013555979,-2.0355967146\H,0,-0.9081556941,-0.0013123641,-0.5858008495\H,0,3.4227700616,-0.0002191626,-0.4593091847\H,0,0.7342398542,0.0016769215,5.7638854239\H,0,3.3085706171,0.0010374073,3.1211064632\H,0,2.1555819002,-0.001260084,-2.3569408242\H,0,-2.3398966622,0.0010127576,5.916620925\H,0,-4.707043599,0.0007285016,6.5922538284\H,0,-3.5283099267,-0.0010290213,1.7414429389\H,0,-6.9021870492,-0.0013774248,2.8925547595

Thermal correction to Enthalpy= 0.231069

Thermal correction to Gibbs Free Energy= 0.166319

Lowest freq: 24.0923

B3LYP TD(nstates=12) /6-311+g(d,p)

HF=-1103.9961136

Excitation energies and oscillator strengths:

Excited State	1:	Singlet-A	2.6857 eV	461.65 nm	f=0.6648
Excited State	2:	Singlet-A	3.0232 eV	410.11 nm	f=0.0000

Excited State 3: Singlet-A 3.1858 eV 389.18 nm f=0.1009
Excited State 4: Singlet-A 3.5566 eV 348.60 nm f=0.0732
Excited State 5: Singlet-A 3.5722 eV 347.08 nm f=0.0000
Excited State 6: Singlet-A 3.6815 eV 336.78 nm f=0.1199
Excited State 7: Singlet-A 3.7569 eV 330.02 nm f=0.0004
Excited State 8: Singlet-A 3.9975 eV 310.15 nm f=0.0082
Excited State 9: Singlet-A 4.0991 eV 302.47 nm f=0.0000
Excited State 10: Singlet-A 4.2516 eV 291.62 nm f=0.0001
Excited State 11: Singlet-A 4.3484 eV 285.12 nm f=0.0000
Excited State 12: Singlet-A 4.4383 eV 279.35 nm f=0.0003

B3LYP TD(nstates=12) /6-311+g(d,p) scrf=(pcm,solvent=water)
Total free energy in solution: with all non-electrostatic terms (a.u.) = -1104.068424
Excitation energies and oscillator strengths:
Excited State 1: Singlet-A 2.7581 eV 449.53 nm f=0.9346
Excited State 2: Singlet-A 3.6115 eV 343.30 nm f=0.0056
Excited State 3: Singlet-A 3.7921 eV 326.95 nm f=0.0762
Excited State 4: Singlet-A 3.8549 eV 321.63 nm f=0.0051
Excited State 5: Singlet-A 4.0713 eV 304.53 nm f=0.0000
Excited State 6: Singlet-A 4.2221 eV 293.65 nm f=0.0401
Excited State 7: Singlet-A 4.2788 eV 289.76 nm f=0.0003
Excited State 8: Singlet-A 4.3816 eV 282.96 nm f=0.0646
Excited State 9: Singlet-A 4.4822 eV 276.62 nm f=0.1234
Excited State 10: Singlet-A 4.5057 eV 275.17 nm f=0.0000
Excited State 11: Singlet-A 4.6235 eV 268.16 nm f=0.0000
Excited State 12: Singlet-A 4.6434 eV 267.01 nm f=0.0035

Appendix A5. Quercetin anion 5–OH

B3LYP/6-31+g(d,p) gas phase

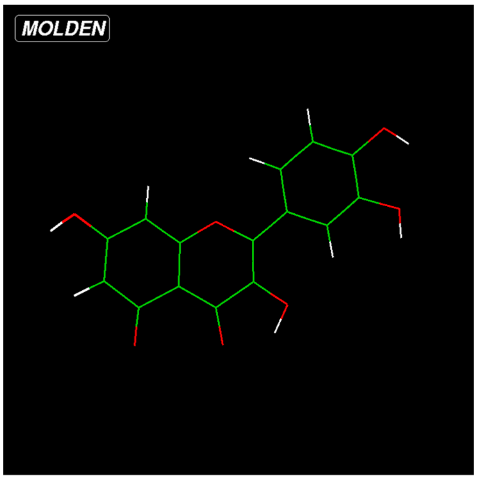

Coordinates:

C,0,0.0070082759,0.0000005073,-0.0122822893\ C,0,0.0043355042,0.0000007195,1.3974073583\ C,0,1.2502801368,0.0000010043,2.0613686278\C,0,2.432346031,0.0000012414,1.3347077248\C,0,2.4269183142,0.0000010882,-0.0658399755\C,0,1.2031352917,0.0000006851,-0.7308845027\C,0,-1.2570171304,0.0000006111,2.1447449839\O,0,-2.3629601802,0.0000000588,1.3268972682\C,0,-3.6471363269,0.0000000077,1.8281324578\C,0,-3.9115247395,0.000000492,3.2271956704\C,0,-2.7920518417,0.0000010801,4.116719814\C,0,-1.4452126261,0.0000010756,3.488783016\C,0,-5.3229650941,0.0000003528,3.6887335676\C,0,-6.3168064862,-0.0000004681,2.6337983933\C,0,-5.9743079475,-0.0000008881,1.2986178931\C,0,-4.6375247523,-0.0000006332,0.8563231461\O,0,-5.647376921,0.0000004574,4.8975761211\O,0,-6.9370820103,-0.0000015968,0.3024245507\O,0,-0.4538011345,0.0000016954,4.4171616818\O,0,-2.7613462749,0.0000011661,5.3734435248\O,0,3.6891135208,0.0000014594,1.924343639\O,0,3.6052140474,0.0000013477,-0.7740016406\H,0,-4.3909124227,-0.0000009872,-0.1977499343\H,0,-7.3590771376,-0.0000007137,2.9477999836\H,0,-1.0108663025,0.0000021027,5.2511118245\H,0,-7.8029395594,-0.0000017148,0.7309884926\H,0,-0.9372595584,0.0000002438,-0.5419793036\H,0,1.1996740055,0.0000005964,-1.8167188629\H,0,1.2791174855,0.0000010498,3.1454839716\H,0,4.3328760566,0.0000021259,-0.1345999328\H,0,3.5914981053,0.0000040841,2.8849904464

Thermal correction to Enthalpy= 0.230403

Thermal correction to Gibbs Free Energy= 0.164233

Lowest freq: 28.5721 cm-1

B3LYP TD(nstates=12) /6-311+g(d,p)

HF=-1103.9633303

Excitation energies and oscillator strengths:

Excited State	1:	Singlet-A	2.4681 eV	502.35 nm	f=0.1015
Excited State	2:	Singlet-A	2.8648 eV	432.78 nm	f=0.0000
Excited State	3:	Singlet-A	2.9066 eV	426.55 nm	f=0.0000

Excited State	4:	Singlet-A	3.1020 eV	399.70 nm	f=0.0087
Excited State	5:	Singlet-A	3.4641 eV	357.91 nm	f=0.0003
Excited State	6:	Singlet-A	3.5946 eV	344.92 nm	f=0.0000
Excited State	7:	Singlet-A	3.7094 eV	334.24 nm	f=0.0005
Excited State	8:	Singlet-A	3.7614 eV	329.62 nm	f=0.1275
Excited State	9:	Singlet-A	3.7702 eV	328.86 nm	f=0.0007
Excited State	10:	Singlet-A	3.8203 eV	324.54 nm	f=0.0000
Excited State	11:	Singlet-A	3.9247 eV	315.91 nm	f=0.3601
Excited State	12:	Singlet-A	3.9550 eV	313.49 nm	f=0.0009

B3LYP TD(nstates=12) /6-311+g(d,p) scrf=(pcm,solvent=water)

Total free energy in solution: with all non-electrostatic terms (a.u.) = -1104.056683

Excitation energies and oscillator strengths:

Excited State	1:	Singlet-A	2.8656 eV	432.66 nm	f=0.2346
Excited State	2:	Singlet-A	3.6346 eV	341.12 nm	f=0.0000
Excited State	3:	Singlet-A	3.7434 eV	331.21 nm	f=0.4524
Excited State	4:	Singlet-A	3.9455 eV	314.24 nm	f=0.0194
Excited State	5:	Singlet-A	4.1921 eV	295.76 nm	f=0.0000
Excited State	6:	Singlet-A	4.4140 eV	280.89 nm	f=0.0160
Excited State	7:	Singlet-A	4.4508 eV	278.57 nm	f=0.0001
Excited State	8:	Singlet-A	4.4771 eV	276.93 nm	f=0.2863
Excited State	9:	Singlet-A	4.6839 eV	264.70 nm	f=0.0002
Excited State	10:	Singlet-A	4.7288 eV	262.19 nm	f=0.0453
Excited State	11:	Singlet-A	4.7683 eV	260.02 nm	f=0.0000
Excited State	12:	Singlet-A	4.8819 eV	253.97 nm	f=0.0000

Appendix A6. Quercetin anion 7–OH

B3LYP/6-31+g(d,p) gas phase

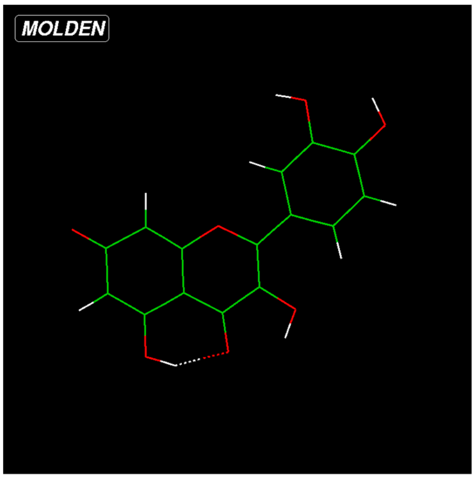

Coordinates:
C,0,0.0147492405,-0.0173296013,-0.0556273427\C,0,-0.0223486398,-0.0021387864,1.3545089644\C,0,1.2068771912,0.0113673486,2.0486823243\C,0,2.4098620535,0.0096554718,1.358136114\C,0,2.4388280722,-0.0054691175,-0.0423685303\C,0,1.2319900081,-0.0188843639,-0.7379894346\C,0,-1.2749328702,0.0001184616,2.116737957\O,0,-1.0590777719,0.0160714034,3.4774797323\C,0,-2.0915176204,0.0211119909,4.3981538263\C,0,-3.4427404264,0.0094126579,3.9310908858\C,0,-3.7065682032,-0.0069713616,2.5511866601\C,0,-2.5539864066,-0.0112551897,1.6468301276\C,0,-4.4823548263,0.0152708526,4.9294005825\C,0,-4.177844572,0.0313749414,6.2653067123\C,0,-2.8012992145,0.0433479655,6.7470823741\C,0,-1.7629378867,0.0372626734,5.7276476334\O,0,-5.7799448363,0.0043335094,4.5080580278\O,0,-2.5269067326,0.0581484597,7.9752741818\O,0,-2.8958396868,-0.0273622346,0.3278622472\ O,0,-4.8636332361,-0.0186597375,1.9981643354\ O,0,3.650407333,0.0224005212,1.9776443387\ O,0,3.6328555396,-0.0070910222,-0.7207530491\H,0,-0.7260048129,0.045806132,6.0459547233\H,0,-4.973248137,0.035581006,7.0036495854\ H,0,-3.8822178449,-0.0316678798,0.373467569\H,0,-5.7648366191,-0.0064008626,3.5203051246\ H,0,-0.9106053731,-0.02789775,-0.6148720144\ H,0,1.2573213084,-0.0305905926,-1.8233765669\ H,0,1.2039237126,0.0231817621,3.1338907031\ H,0,4.3458643475,0.0035078203,-0.0650108772\H,0,3.5342358582,0.032327257,2.9361430314

Thermal correction to Enthalpy= 0.231289

Thermal correction to Gibbs Free Energy= 0.165862

Lowest freq: 24.2265 cm-1

B3LYP TD(nstates=12) /6-311+g(d,p)

HF=-1103.9866386

Excitation energies and oscillator strengths:

Excited State	1:	Singlet-A	2.5961 eV	477.58 nm	f=0.2410
Excited State	2:	Singlet-A	3.0237 eV	410.04 nm	f=0.0000
Excited State	3:	Singlet-A	3.0710 eV	403.73 nm	f=0.0000

Excited State	4:	Singlet-A	3.2034 eV	387.04 nm	f=0.0226
Excited State	5:	Singlet-A	3.3339 eV	371.89 nm	f=0.0064
Excited State	6:	Singlet-A	3.8239 eV	324.23 nm	f=0.0004
Excited State	7:	Singlet-A	3.8425 eV	322.67 nm	f=0.0000
Excited State	8:	Singlet-A	3.9005 eV	317.87 nm	f=0.0003
Excited State	9:	Singlet-A	3.9639 eV	312.78 nm	f=0.3151
Excited State	10:	Singlet-A	3.9876 eV	310.93 nm	f=0.0001
Excited State	11:	Singlet-A	4.1239 eV	300.65 nm	f=0.0163
Excited State	12:	Singlet-A	4.1785 eV	296.72 nm	f=0.0000

B3LYP TD(nstates=12) /6-311+g(d,p) scrf=(pcm,solvent=water)

Total free energy in solution: with all non-electrostatic terms (a.u.) = -1104.065982

Excitation energies and oscillator strengths:

Excited State	1:	Singlet-A	2.9687 eV	417.64 nm	f=0.4815
Excited State	2:	Singlet-A	3.7208 eV	333.22 nm	f=0.0653
Excited State	3:	Singlet-A	3.8406 eV	322.83 nm	f=0.2465
Excited State	4:	Singlet-A	3.9650 eV	312.69 nm	f=0.0000
Excited State	5:	Singlet-A	4.1957 eV	295.50 nm	f=0.0221
Excited State	6:	Singlet-A	4.4278 eV	280.01 nm	f=0.0087
Excited State	7:	Singlet-A	4.5948 eV	269.84 nm	f=0.3865
Excited State	8:	Singlet-A	4.6033 eV	269.34 nm	f=0.0008
Excited State	9:	Singlet-A	4.7162 eV	262.89 nm	f=0.0000
Excited State	10:	Singlet-A	4.7673 eV	260.07 nm	f=0.0105
Excited State	11:	Singlet-A	4.8212 eV	257.16 nm	f=0.0005
Excited State	12:	Singlet-A	4.8861 eV	253.75 nm	f=0.0107

Appendix A7. Quercetin radical 3–OH

B3LYP/6-31+g(d,p) gas phase

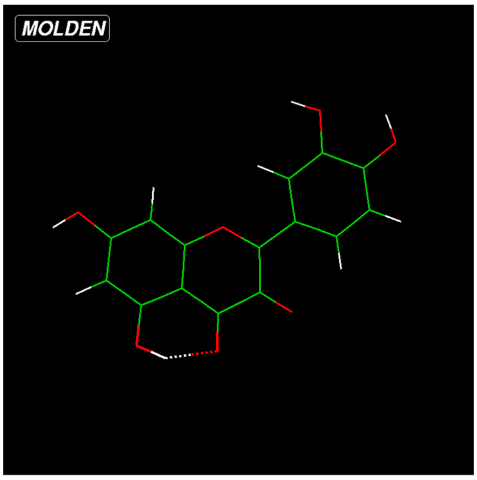

Coordinates:
C,0,0.0022645505,-0.0121847062,0.0168201966\ C,0,0.002905019,0.0012461642,1.4393750599\ C,0,1.2541423258,0.0166486978,2.1081728467\C,0,2.4423182665,0.0184926785,1.3889763055\C,0,2.4288157968,0.0052142902,-0.0068439213\C,0,1.1902548713,-0.0101832531,-0.6848271011\C,0,-1.2536371148,-0.0011661434,2.1486139635\C,0,-1.438252584,0.0109055973,3.5874392409\C,0,-2.8621038168,0.0055856891,4.1073232348\C,0,-3.9208958446,-0.0111532808,3.1264544891\C,0,-3.6279382111,-0.0218920999,1.7512074544\O,0,-2.3320369908,-0.0167110367,1.3122801431\C,0,-5.2927634622,-0.0173080809,3.5189128327\C,0,-6.3003116601,-0.0335305361,2.552640384\C,0,-5.9508931091,-0.0436719743,1.1979608616\C,0,-4.6127311872,-0.0380415945,0.775662585\O,0,-5.6223817543,-0.0076000703,4.8136728284\O,0,-6.8903997788,-0.0595713946,0.2141193903\O,0,-3.0844504587,0.0154506353,5.3306031747\O,0,-0.4920410014,0.025157112,4.3931753556\ O,0,3.5932994619,0.0071571577,-0.6969765376\O,0,1.2906133063,-0.0224189915,-2.0576079894\H,0,-4.3661035187,-0.0461087989,-0.2783710106\H,0,-7.337724674,-0.0379700698,2.872530788\H,0,-4.7688918017,0.0033292578,5.3319465829\H,0,-7.7779931766,-0.0623523032,0.5976870281\ H,0,1.2725176918,0.0269713624,3.1883756711\H,0,3.3996494738,0.0302653555,1.8989631933\H,0,-0.9387746628,-0.0241053051,-0.5219402709\H,0,0.4168059286,-0.0326776514,-2.4687317584\ H,0,3.3985772657,-0.003241257,-1.6476225956

Thermal correction to Enthalpy= 0.231720
Thermal correction to Gibbs Free Energy= 0.165424
Lowest freq: 26.8366 cm-1

B3LYP TD(nstates=10) /6-311+g(d,p)
HF=-1103.872719

Excited State	1:	?Spin	-A	1.4680 eV	844.58 nm	f=0.0000
Excited State	2:	?Spin	-A	2.0289 eV	611.10 nm	f=0.0465
Excited State	3:	?Spin	-A	2.2711 eV	545.92 nm	f=0.0467

Excited State 4: ?Spin -A 2.4399 eV 508.16 nm f=0.0141
Excited State 5: ?Spin -A 2.6188 eV 473.44 nm f=0.2419
Excited State 6: ?Spin -A 2.8032 eV 442.30 nm f=0.0910
Excited State 7: ?Spin -A 2.8048 eV 442.04 nm f=0.0000
Excited State 8: ?Spin -A 3.2760 eV 378.46 nm f=0.0000
Excited State 9: ?Spin -A 3.3354 eV 371.72 nm f=0.0126
Excited State 10: ?Spin -A 3.5145 eV 352.78 nm f=0.0115

B3LYP TD(nstates=10) /6-311+g(d,p) scrf=(pcm,solvent=water)
Total free energy in solution: with all non-electrostatic terms (a.u.) = -1103.902902
Excited State 1: ?Spin -A 1.7206 eV 720.57 nm f=0.0000
Excited State 2: ?Spin -A 1.7599 eV 704.51 nm f=0.0904
Excited State 3: ?Spin -A 2.0962 eV 591.46 nm f=0.0441
Excited State 4: ?Spin -A 2.1795 eV 568.87 nm f=0.0197
Excited State 5: ?Spin -A 2.4794 eV 500.06 nm f=0.2632
Excited State 6: ?Spin -A 2.6003 eV 476.81 nm f=0.1350
Excited State 7: ?Spin -A 3.0270 eV 409.60 nm f=0.0000
Excited State 8: ?Spin -A 3.2402 eV 382.64 nm f=0.0171
Excited State 9: ?Spin -A 3.4092 eV 363.67 nm f=0.0110
Excited State 10: ?Spin -A 3.5579 eV 348.47 nm f=0.0000

Appendix A8. Quercetin radical 3′–OH

B3LYP/6-31+g(d,p) gas phase

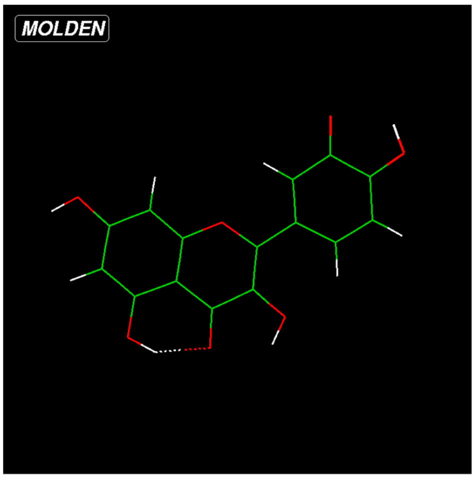

Coordinates:

C,0,-0.0002823267,0.0822717355,0.0023356495\ C,0,0.000317777,0.0307703469,1.3909834114\ C,0,1.1925579355,-0.0226210174,2.1412605207\C,0,2.4371136945,-0.0236052177,1.4472093477\ C,0,2.4537628638,0.0275368655,0.058130848\C,0,1.2385875782,0.079734487,-0.6463554621\C,0,1.1182821596,-0.0740085898,3.5694256587\C,0,-0.2119969597,-0.0675077695,4.1636308966\C,0,-1.3396733059,-0.014489903,3.3855803047\O,0,-1.2074449097,0.0334293064,2.0187320873\C,0,-2.746764078,0.0001393918,3.802992486\C,0,-3.1044393973,-0.0437766307,5.194322019\C,0,-4.4291561688,-0.0309733426,5.613142311\C,0,-5.4434548497,0.0259851214,4.6612933651\C,0,-5.1348215316,0.0724559377,3.2290811866\C,0,-3.7516005788,0.0570380385,2.8471779276\O,0,-6.1062203061,0.1229988842,2.4258981277\O,0,-6.7339445988,0.0414242249,4.9890723904\O,0,2.1178696724,-0.123927287,4.3372621764\O,0,-0.2246163769,-0.11746132,5.5175751512\O,0,3.5937775029,-0.0736310969,2.1270198598\ O,0,1.2095786776,0.1307152768,-2.0051678894\H,0,-0.9232852706,0.1229864853,-0.5618368176\H,0,3.4076271169,0.0262784083,-0.4603564532\H,0,0.721952348,-0.1472851867,5.7737182805\H,0,3.3786939779,-0.1048192471,3.092464504\H,0,2.1080765495,0.1247368874,-2.3620105368\H,0,-2.3210074344,-0.0880668558,5.9375873484\H,0,-4.6786202858,-0.0647522903,6.6683397862\H,0,-3.5301016262,0.0915273471,1.7884799316\H,0,-7.2179285931,0.083483487,4.1334189056

Thermal correction to Enthalpy= 0.232352
Thermal correction to Gibbs Free Energy= 0.166151
Lowest freq: 7.9271 cm-1

B3LYP TD(nstates=10) /6-311+g(d,p)
Excitation energies and oscillator strengths:

Excited State 1: ?Spin -A 1.4535 eV 852.98 nm f=0.0220

Excited State 2: ?Spin -A 1.7262 eV 718.24 nm f=0.0000
Excited State 3: ?Spin -A 2.0860 eV 594.36 nm f=0.0036
Excited State 4: ?Spin -A 2.4422 eV 507.68 nm f=0.0270
Excited State 5: ?Spin -A 2.6265 eV 472.06 nm f=0.0034
Excited State 6: ?Spin -A 2.8822 eV 430.17 nm f=0.0146
Excited State 7: ?Spin -A 3.0836 eV 402.07 nm f=0.1818
Excited State 8: ?Spin -A 3.2987 eV 375.85 nm f=0.0441
Excited State 9: ?Spin -A 3.4409 eV 360.33 nm f=0.0000
Excited State 10: ?Spin -A 3.4443 eV 359.97 nm f=0.0430

B3LYP TD(nstates=10) /6-311+g(d,p) scrf=(pcm,solvent=water)
Total free energy in solution with all non-electrostatic terms (a.u.) = -1103.898842
Excitation energies and oscillator strengths:
Excited State 1: ?Spin -A 1.2962 eV 956.48 nm f=0.0357
Excited State 2: ?Spin -A 1.8981 eV 653.19 nm f=0.0000
Excited State 3: ?Spin -A 1.9102 eV 649.08 nm f=0.0075
Excited State 4: ?Spin -A 2.3564 eV 526.16 nm f=0.0209
Excited State 5: ?Spin -A 2.4625 eV 503.50 nm f=0.0131
Excited State 6: ?Spin -A 2.7187 eV 456.04 nm f=0.0527
Excited State 7: ?Spin -A 3.0902 eV 401.21 nm f=0.2634
Excited State 8: ?Spin -A 3.2873 eV 377.16 nm f=0.0450
Excited State 9: ?Spin -A 3.3403 eV 371.18 nm f=0.0000
Excited State 10: ?Spin -A 3.3933 eV 365.38 nm f=0.0098

Appendix A9. Quercetin radical 4′–OH

B3LYP/6-31+g(d,p) gas phase

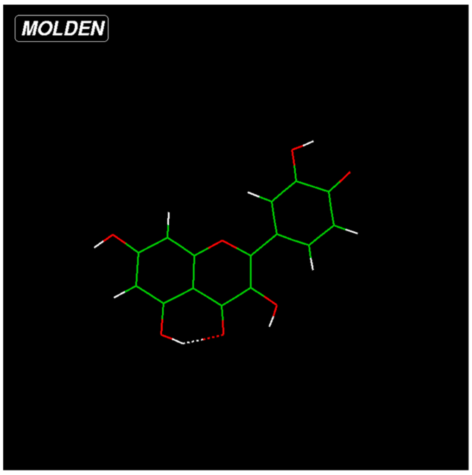

Coordinates:

C,0,0.0092101311,-0.0008555204,-0.0093248652\C,0,0.0024838779,-0.0002989597,1.3793654059\C,0,1.1928589152,0.0002846902,2.1358058229\C,0,2.441185289,0.0003017259,1.4477186148\C,0,2.4643922406,-0.0002517098,0.0577947066\C,0,1.2519255574,-0.0008219489,-0.6526420358\C,0,1.1129243484,0.0008410215,3.563181981\C,0,-0.2200211872,0.0007662633,4.1536583581\C,0,-1.3567408955,0.0001807087,3.3685383074\O,0,-1.2091132914,-0.0003369092,1.9993394622\C,0,-2.7459269071,0.0000162718,3.7801686255\C,0,-3.1130305868,0.0005176952,5.1732079326\C,0,-4.4246999737,0.0003674256,5.5635893811\C,0,-5.4898933404,-0.0003020351,4.5891247262\C,0,-5.0855987032,-0.0008041164,3.174969328\C,0,-3.7599178335,-0.0006468783,2.7927340223\O,0,-6.0826895661,-0.0014236827,2.2823653829\O,0,-6.7158820431,-0.0004854316,4.8513529602\O,0,2.1076834463,0.0013865424,4.3392252315\O,0,-0.2328990055,0.0013052162,5.5018971009\O,0,3.5947452168,0.0008484558,2.1336107047\O,0,1.2272093183,-0.0013738391,-2.0115957629\H,0,-0.9109565851,-0.0013007338,-0.5795194215\H,0,3.4206865185,-0.0002335195,-0.4560608911\H,0,0.7150071818,0.0016313896,5.7601445122\H,0,3.3775497367,0.0011857416,3.0986995223\H,0,2.1267595723,-0.0013038059,-2.366083338\H,0,-2.3338269099,0.0010229061,5.9216983396\H,0,-4.7028460706,0.0007455917,6.6123148121\H,0,-3.5107580543,-0.0010333619,1.739829707\H,0,-6.9082580061,-0.0013967575,2.8143496621

Thermal correction to Enthalpy= 0.232345
Thermal correction to Gibbs Free Energy= 0.167690
Lowest freq: 21.0365 cm-1

B3LYP TD(nstates=10) /6-311+g(d,p)
HF=-1103.8861223
Excitation energies and oscillator strengths:

Excited State	1:	?Spin	-A	1.7086 eV	725.67 nm	f=0.0486
Excited State	2:	?Spin	-A	1.7913 eV	692.16 nm	f=0.0000

Excited State	3:	?Spin	-A	1.9384 eV	639.61 nm	f=0.0061
Excited State	4:	?Spin	-A	2.4471 eV	506.65 nm	f=0.0331
Excited State	5:	?Spin	-A	2.5715 eV	482.14 nm	f=0.0050
Excited State	6:	?Spin	-A	2.9153 eV	425.29 nm	f=0.2909
Excited State	7:	?Spin	-A	3.1749 eV	390.51 nm	f=0.2105
Excited State	8:	?Spin	-A	3.2257 eV	384.36 nm	f=0.0000
Excited State	9:	?Spin	-A	3.5639 eV	347.89 nm	f=0.0064
Excited State	10:	?Spin	-A	3.6727 eV	337.59 nm	f=0.0019

B3LYP TD(nstates=10) /6-311+g(d,p) scrf=(pcm,solvent=water)
Total free energy in solution: with all non-electrostatic terms (a.u.) = -1103.903187
Excitation energies and oscillator strengths:

Excited State	1:	?Spin	-A	1.5323 eV	809.14 nm	f=0.0854
Excited State	2:	?Spin	-A	1.8552 eV	668.29 nm	f=0.0062
Excited State	3:	?Spin	-A	2.0000 eV	619.92 nm	f=0.0000
Excited State	4:	?Spin	-A	2.2522 eV	550.51 nm	f=0.0398
Excited State	5:	?Spin	-A	2.4046 eV	515.61 nm	f=0.0011
Excited State	6:	?Spin	-A	2.8077 eV	441.58 nm	f=0.3792
Excited State	7:	?Spin	-A	3.1333 eV	395.69 nm	f=0.2278
Excited State	8:	?Spin	-A	3.1348 eV	395.51 nm	f=0.0000
Excited State	9:	?Spin	-A	3.5110 eV	353.13 nm	f=0.0042
Excited State	10:	?Spin	-A	3.5818 eV	346.15 nm	f=0.0033

Appendix A10. Quercetin radical 5–OH

B3LYP/6-31+g(d,p) gas phase

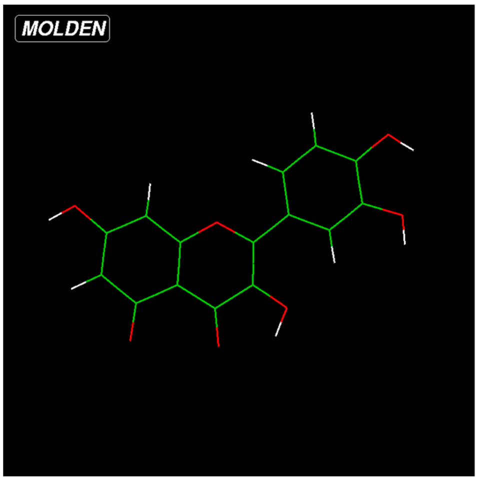

Coordinates:
C,0,-0.0179676269,0.0000005829,0.006558922\C,0,-0.0002412969,0.0000006139,1.4165295601\C,0,1.2512271688,0.0000005907,2.0739671946\C,0,2.424634518,0.0000009065,1.3369825476\C,0,2.3978761406,0.0000009521,-0.0689474441\C,0,1.1665828435,0.0000007292,-0.7237402191\C,0,-1.2513632181,0.0000005158,2.1711482375\O,0,-2.3640082591,-0.0000000898,1.3659417315\C,0,-3.6240454314,-0.0000001595,1.8677237247\C,0,-3.8961573668,0.0000003205,3.2357639368\C,0,-2.7764656015,0.0000009156,4.156870578\C,0,-1.4441669449,0.0000009922,3.5294056124\C,0,-5.3121693111,0.0000002414,3.656656236\C,0,-6.3237688313,-0.0000003868,2.608803071\C,0,-5.982951745,-0.0000008614,1.2693400575\C,0,-4.6310076365,-0.0000007558,0.8799224629\O,0,-5.6584629862,0.0000006845,4.8522319123\O,0,-6.8937948881,-0.0000014484,0.2555225152\O,0,-0.4403113656,0.0000016392,4.4270919978\O,0,-2.7996456911,0.0000013931,5.3985730853\O,0,3.6854119834,0.000000543,1.8913056108\O,0,3.5503751316,0.0000012208,-0.7882735682\ H,0,-4.3593899237,-0.00000113,-0.1690355286\H,0,-7.3576649038,-0.0000004529,2.9418021641\ H,0,-0.922624878,0.0000018056,5.2929484592\H,0,-7.7926985172,-0.0000014914,0.6120138343\ H,0,-0.9633363503,0.0000005182,-0.5204643182\H,0,1.1541488382,0.0000008296,-1.8085469943\H,0,1.2970407951,0.00000029,3.1560421263\ H,0,4.3021893554,0.0000021791,-0.1753944278\H,0,3.6361043276,0.0000065769,2.8559546381

Thermal correction to Enthalpy= 0.231008
Thermal correction to Gibbs Free Energy= 0.164050
Lowest freq: 22.2251 cm-1

B3LYP TD(nstates=10) /6-311+g(d,p)
HF=-1103.8505164
Excitation energies and oscillator strengths:

Excited State	1:	?Spin	-A	0.8972 eV	1381.97 nm	f=0.0000
Excited State	2:	?Spin	-A	1.1507 eV	1077.48 nm	f=0.0865

Excited State 3: ?Spin -A 1.6404 eV 755.80 nm f=0.0039
Excited State 4: ?Spin -A 2.0347 eV 609.35 nm f=0.0080
Excited State 5: ?Spin -A 2.3408 eV 529.66 nm f=0.0000
Excited State 6: ?Spin -A 2.4542 eV 505.19 nm f=0.0011
Excited State 7: ?Spin -A 2.4671 eV 502.55 nm f=0.0085
Excited State 8: ?Spin -A 3.1651 eV 391.73 nm f=0.0165
Excited State 9: ?Spin -A 3.4936 eV 354.89 nm f=0.0061
Excited State 10: ?Spin -A 3.5161 eV 352.62 nm f=0.0000

B3LYP TD(nstates=10) /6-311+g(d,p) scrf=(pcm,solvent=water)

Total free energy in solution: with all non-electrostatic terms (a.u.) = -1103.883714

Excitation energies and oscillator strengths:

Excited State 1: ?Spin -A 1.0227 eV 1212.36 nm f=0.1096
Excited State 2: ?Spin -A 1.2817 eV 967.37 nm f=0.0000
Excited State 3: ?Spin -A 1.5531 eV 798.32 nm f=0.0007
Excited State 4: ?Spin -A 1.8028 eV 687.75 nm f=0.0127
Excited State 5: ?Spin -A 2.3143 eV 535.74 nm f=0.0009
Excited State 6: ?Spin -A 2.4664 eV 502.69 nm f=0.0123
Excited State 7: ?Spin -A 2.6778 eV 463.00 nm f=0.0000
Excited State 8: ?Spin -A 3.0847 eV 401.93 nm f=0.0266
Excited State 9: ?Spin -A 3.3649 eV 368.46 nm f=0.2723
Excited State 10: ?Spin -A 3.4338 eV 361.07 nm f=0.2349

Appendix A11. Quercetin radical 7–OH

B3LYP/6-31+g(d,p) gas phase

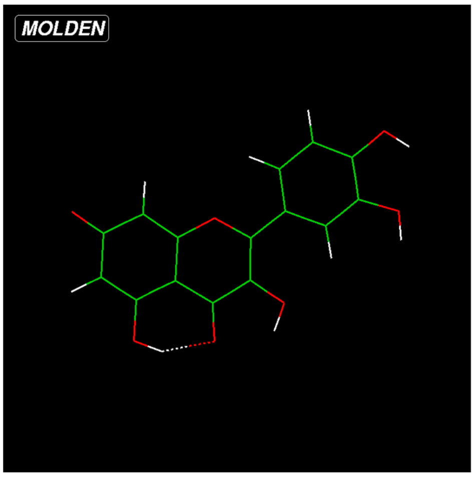

Coordinates:

C,0,0.0102158452,-0.0000000649,-0.0085169555\ C,0,0.0052283676,0.0000003202,1.4034307547\ C,0,1.2469781412,0.0000005327,2.0824972793\C,0,2.4311053901,0.0000009877,1.3652529478\C,0,2.4257343345,0.000000785,-0.0425414786\C,0,1.2047195987,0.0000001435,-0.7187764697\C,0,-1.2609098554,0.0000002698,2.1266857979\O,0,-2.3466767022,-0.0000002854,1.2984652978\C,0,-3.6341189829,-0.00000036,1.7592818498\C,0,-3.9050407191,-0.0000000149,3.146448581\C,0,-2.7993880776,0.0000005325,4.0645796554\C,0,-1.4718053836,0.0000007596,3.4917724966\C,0,-5.2759950554,0.0000000384,3.6022259877\C,0,-6.3032446174,-0.0000001204,2.6896093881\C,0,-6.0317424915,-0.0000004758,1.266989722\C,0,-4.6450131629,-0.0000006371,0.819642309\ O,0,-5.531750896,-0.0000000559,4.9233566985\ O,0,-6.9565276095,-0.0000006805,0.4146165914\ O,0,-0.4673947316,0.0000014759,4.3983255514\O,0,-2.9107998648,0.0000020488,5.3230116503\ O,0,3.6834092921,0.0000007207,1.9351404204\O,0,3.5863633587,0.0000011809,-0.7414019553\H,0,-4.4383336425,-0.0000008793,-0.2439377265\H,0,-7.3360734989,-0.0000001072,3.0173691475\H,0,-0.9240699184,0.0000013155,5.2684029868\H,0,-4.6717146779,-0.0000006576,5.4092683471\H,0,-0.9269975154,-0.0000003825,-0.549419782\H,0,1.2105073297,0.0000000376,-1.8034771602\ H,0,1.2773485864,0.0000003302,3.1646167105\H,0,4.3306265541,0.0000024865,-0.1187638906\H,0,3.6251923661,0.0000094019,2.8993367593

Thermal correction to Enthalpy= 0.231227
Thermal correction to Gibbs Free Energy= 0.165045
Lowest freq: 19.2127 cm-1

B3LYP TD(nstates=10) /6-311+g(d,p)
HF=-1103.8638607
Excitation energies and oscillator strengths:

Excited State 1: ?Spin -A 0.8594 eV 1442.68 nm f=0.0000

Excited State 2: ?Spin -A 0.9985 eV 1241.73 nm f=0.0296
Excited State 3: ?Spin -A 1.4043 eV 882.88 nm f=0.0729
Excited State 4: ?Spin -A 2.1218 eV 584.33 nm f=0.0001
Excited State 5: ?Spin -A 2.4581 eV 504.40 nm f=0.0516
Excited State 6: ?Spin -A 2.5166 eV 492.67 nm f=0.0045
Excited State 7: ?Spin -A 2.7110 eV 457.34 nm f=0.0000
Excited State 8: ?Spin -A 3.1095 eV 398.73 nm f=0.0246
Excited State 9: ?Spin -A 3.1393 eV 394.94 nm f=0.0560
Excited State 10: ?Spin -A 3.3450 eV 370.65 nm f=0.2186

B3LYP TD(nstates=10) /6-311+g(d,p) scrf=(pcm,solvent=water)
Total free energy in solution: with all non-electrostatic terms (a.u.) = -1103.884922
Excitation energies and oscillator strengths:
Excited State 1: ?Spin -A 0.9484 eV 1307.36 nm f=0.0724
Excited State 2: ?Spin -A 1.1062 eV 1120.81 nm f=0.0976
Excited State 3: ?Spin -A 1.2775 eV 970.50 nm f=0.0000
Excited State 4: ?Spin -A 1.7217 eV 720.12 nm f=0.0096
Excited State 5: ?Spin -A 2.2398 eV 553.56 nm f=0.0126
Excited State 6: ?Spin -A 2.5197 eV 492.07 nm f=0.0634
Excited State 7: ?Spin -A 2.6264 eV 472.07 nm f=0.0000
Excited State 8: ?Spin -A 2.9876 eV 415.00 nm f=0.0302
Excited State 9: ?Spin -A 3.1408 eV 394.75 nm f=0.1121
Excited State 10: ?Spin -A 3.2341 eV 383.37 nm f=0.2431

Appendix A12. Quercetin anion 3′–OH to 4′–OH

B3LYP/6-31+g(d,p) gas phase

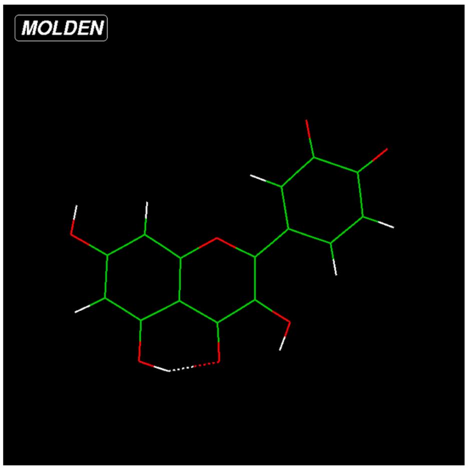

Coordinates:
C,0,-0.0117431408,-0.0000487222,0.0053542299\C,0,-0.0091392746,0.0000185989,1.4035331873\C,0,1.1832744519,0.0000398727,2.1392725666\C,0,2.42132764,-0.0000026424,1.4405989568\C,0,2.4391152118,-0.0000747267,0.0481088008\C,0,1.2222124245,-0.0000962196,-0.6485847204\O,0,-1.2097527278,0.000061097,2.0308632368\ C,0,-1.3536221205,0.0001171425,3.4094206165\ C,0,-0.187827859,0.0001271574,4.1711151868\ C,0,1.1099561676,0.0000964462,3.5787513995\C,0,-2.7337823679,0.0001372286,3.8213514908\C,0,-3.7627964155,0.0000200302,2.8683988261\C,0,-5.1588421419,0.0000374682,3.2057228967\C,0,-5.521367539,0.000220832,4.6857234775\C,0,-4.4029902863,0.0003246987,5.6199319921\ C,0,-3.0963882986,0.0002873006,5.2224638569\O,0,-6.7030543314,0.0002424855,5.0687864524\O,0,-6.0535717202,-0.000055804,2.3254719554\ O,0,-0.1919521274,0.0001563773,5.539286636\ O,0,2.1507935157,0.0000724143,4.325033592\O,0,3.5767897035,0.0000346608,2.1353137891\O,0,1.3024451473,-0.0001625569,-2.0197674171\H,0,-0.954003291,-0.000063897,-0.5333647168\ H,0,3.3789494489,-0.0001080336,-0.4910272067\ H,0,0.759384302,0.0001574887,5.7715023424\ H,0,3.3226004228,0.0001196365,3.1025713165\H,0,0.4107723416,-0.0001715643,-2.3916728969\H,0,-2.3109516544,0.0003769903,5.9661565366\ H,0,-4.6591219246,0.0004426614,6.6768111006\H,0,-3.5352082238,-0.0000950836,1.8088661602

Thermal correction to Enthalpy= 0.218600
Thermal correction to Gibbs Free Energy= 0.152517
Lowest freq: 16.8973 cm-1

B3LYP TD(nstates=10) /6-311+g(d,p)
HF=-1103.3600338
Excitation energies and oscillator strengths:

Excited State 1: ?Spin -A 1.3154 eV 942.56 nm f=0.0000

Excited State 2: ?Spin -A 1.6689 eV 742.91 nm f=0.1236
Excited State 3: ?Spin -A 1.8870 eV 657.06 nm f=0.1083
Excited State 4: ?Spin -A 2.2898 eV 541.46 nm f=0.0000
Excited State 5: ?Spin -A 2.5623 eV 483.88 nm f=0.0086
Excited State 6: ?Spin -A 2.6738 eV 463.70 nm f=0.1056
Excited State 7: ?Spin -A 2.6763 eV 463.26 nm f=0.0000
Excited State 8: ?Spin -A 2.7888 eV 444.57 nm f=0.0000
Excited State 9: ?Spin -A 2.9975 eV 413.63 nm f=0.0000
Excited State 10: ?Spin -A 3.0330 eV 408.79 nm f=0.0029

B3LYP TD(nstates=10) /6-311+g(d,p) scrf=(pcm,solvent=water)
Total free energy in solution: with all non-electrostatic terms (a.u.) = -1103.439710
Excitation energies and oscillator strengths:
Excited State 1: ?Spin -A 1.6500 eV 751.44 nm f=0.0000
Excited State 2: ?Spin -A 1.6761 eV 739.71 nm f=0.0361
Excited State 3: ?Spin -A 1.9994 eV 620.11 nm f=0.1574
Excited State 4: ?Spin -A 2.4191 eV 512.52 nm f=0.1981
Excited State 5: ?Spin -A 2.6403 eV 469.58 nm f=0.0000
Excited State 6: ?Spin -A 2.8533 eV 434.53 nm f=0.0118
Excited State 7: ?Spin -A 3.0172 eV 410.92 nm f=0.0486
Excited State 8: ?Spin -A 3.0760 eV 403.07 nm f=0.0468
Excited State 9: ?Spin -A 3.3382 eV 371.42 nm f=0.0480
Excited State 10: ?Spin -A 3.3643 eV 368.53 nm f=0.0708

Appendix A13. Quercetin radical anion 3′–OH to 5–OH

B3LYP/6-31+g(d,p) gas phase

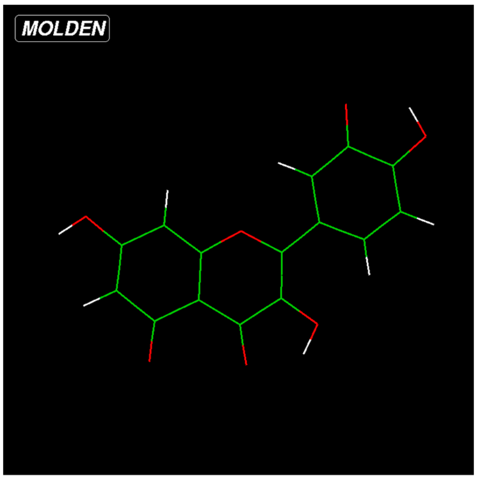

Coordinates:
C,0,0.0208938851,-0.0001060171,-0.0430109861\ C,0,0.0094514269,0.0000646029,1.390987054\ C,0,1.2202288494,0.0001037244,2.0915475122\C,0,2.4665182289,-0.0000225592,1.4067865238\ C,0,2.419145524,-0.0001940979,-0.0562015584\C,0,1.2169924108,-0.0002335912,-0.7550739758\C,0,-1.2588605554,0.0001959224,2.1097781269\O,0,-1.1006626403,0.0003601898,3.4723750692\C,0,-2.1595974545,0.000503965,4.3460393632\C,0,-3.5064126447,0.0004915466,3.8997341503\C,0,-3.742343601,0.0003255911,2.4904022516\C,0,-2.5388090599,0.000177698,1.6181489519\C,0,-4.5912883941,0.0006510188,4.912268801\C,0,-4.1591196085,0.0008107497,6.2973232583\C,0,-2.8276574293,0.0008120825,6.6473577726\C,0,-1.7954226841,0.0006588963,5.6852640223\O,0,-5.803081932,0.0006516545,4.6086393089\O,0,-2.4144794427,0.0009627221,7.9637578521\O,0,-2.8747029994,0.0000281162,0.3149188062\O,0,-4.8267049704,0.0002765161,1.858043574\ O,0,3.6277986546,-0.0000042311,1.9301032713\O,0,3.6205941127,-0.0003085444,-0.6542920232\H,0,-0.7538972003,0.000662235,5.9802824\H,0,-4.9437459099,0.0009295373,7.0506383752\H,0,-3.875730431,0.0000765126,0.394734349\H,0,-3.1994960197,0.0010604892,8.5270269675\H,0,-0.918411435,-0.000135236,-0.5779088683\H,0,1.2195355454,-0.0003619641,-1.8409665199\ H,0,1.2343816994,0.0002308291,3.173777685\H,0,4.2389487489,-0.0002372643,0.1210958482

Thermal correction to Enthalpy= 0.217911
Thermal correction to Gibbs Free Energy= 0.152097
lowest freq: 23.0434 cm-1

B3LYP TD(nstates=10) /6-311+g(d,p)
HF=-1103.3432118
Excitation energies and oscillator strengths:

Excited State	1:	?Spin	-A	0.6467 eV	1917.25 nm	f=0.1066
Excited State	2:	?Spin	-A	1.0852 eV	1142.54 nm	f=0.0000
Excited State	3:	?Spin	-A	1.3418 eV	924.05 nm	f=0.0117

Excited State 4: ?Spin -A 1.7802 eV 696.44 nm f=0.0009
Excited State 5: ?Spin -A 1.9172 eV 646.70 nm f=0.0000
Excited State 6: ?Spin -A 2.3808 eV 520.77 nm f=0.0192
Excited State 7: ?Spin -A 2.4136 eV 513.70 nm f=0.0016
Excited State 8: ?Spin -A 2.5906 eV 478.59 nm f=0.0000
Excited State 9: ?Spin -A 2.6746 eV 463.56 nm f=0.1014
Excited State 10: ?Spin -A 3.0289 eV 409.34 nm f=0.0008

B3LYP TD(nstates=10) /6-311+g(d,p) scrf=(pcm,solvent=water)
Total free energy in solution: with all non-electrostatic terms (a.u.) = -1103.420107
Excitation energies and oscillator strengths:
Excited State 1: ?Spin -A 0.6982 eV 1775.88 nm f=0.0971
Excited State 2: ?Spin -A 1.3670 eV 906.96 nm f=0.0128
Excited State 3: ?Spin -A 1.7194 eV 721.07 nm f=0.0000
Excited State 4: ?Spin -A 1.9171 eV 646.73 nm f=0.0059
Excited State 5: ?Spin -A 2.0342 eV 609.50 nm f=0.0000
Excited State 6: ?Spin -A 2.2932 eV 540.66 nm f=0.0098
Excited State 7: ?Spin -A 2.3619 eV 524.94 nm f=0.0661
Excited State 8: ?Spin -A 2.7600 eV 449.22 nm f=0.1276
Excited State 9: ?Spin -A 3.0409 eV 407.72 nm f=0.0098
Excited State 10: ?Spin -A 3.0493 eV 406.60 nm f=0.0000

Appendix A14. Quercetin radical anion 4′–OH to 7–OH

B3LYP/6-31+g(d,p) gas phase

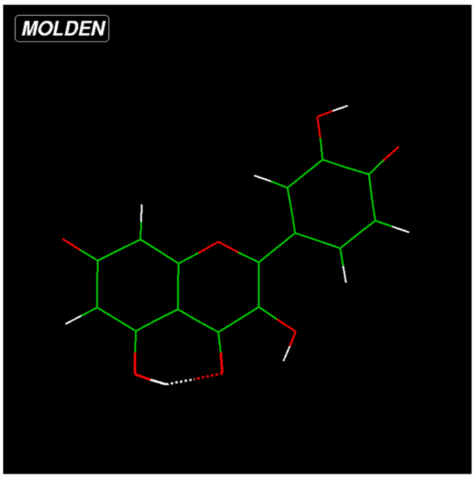

Coordinates:

C,0,0.0192995846,-0.0008474387,-0.0137876706\ C,0,0.0011761707,-0.0003154627,1.3546039336\ C,0,1.194190604,0.000200514,2.1376332263\C,0,2.4593847742,0.0001455181,1.4412434222\C,0,2.5036634568,-0.0003779673,0.0726191337\C,0,1.2890700691,-0.0009032606,-0.7319292228\ C,0,1.1043848282,0.000756363,3.5437620118\ C,0,-0.2271169172,0.0007351792,4.1254040618\ C,0,-1.3790241465,0.0002045104,3.3473047172\O,0,-1.226484487,-0.0002857727,1.9821612612\C,0,-2.7497802402,0.0000347603,3.7719516145\C,0,-3.1111352043,0.0008041244,5.1601460352\C,0,-4.42586368,0.0006759153,5.5606912679\C,0,-5.4991674914,-0.0002373841,4.6043435814\C,0,-5.1043532429,-0.0010072391,3.1945766471\C,0,-3.7914192381,-0.0008688736,2.7964254148\O,0,-6.1347654823,-0.0018703615,2.3161471337\O,0,-6.7333966567,-0.00042922,4.8765300535\ O,0,2.090726131,0.0012306178,4.3639051968\ O,0,-0.2188735353,0.0012359341,5.4760581591\ O,0,3.6000415231,0.0006237961,2.1791256789\O,0,1.3271234365,-0.0013851414,-1.986816498\H,0,-0.9004077124,-0.0012364953,-0.5873294984\ H,0,3.4568236754,-0.0004136008,-0.4449105205\ H,0,0.7504655324,0.0014884607,5.6780585637\ H,0,3.3357105548,0.0009558376,3.1329694207\H,0,-2.3280161122,0.001508053,5.9053614566\ H,0,-4.6900212237,0.0012771501,6.6139887044\ H,0,-3.5498905332,-0.001454853,1.7410049909\ H,0,-6.9294839526,-0.0017382333,2.8943638689

Thermal correction to Enthalpy= 0.218739
Thermal correction to Gibbs Free Energy= 0.153791
Lowest freq: 26.3164 cm-1

B3LYP TD(nstates=10) /6-311+g(d,p)
HF=-1103.375027
Excitation energies and oscillator strengths:

Excited State	1:	?Spin	-A	1.0299 eV	1203.86 nm	f=0.1725
Excited State	2:	?Spin	-A	1.3901 eV	891.92 nm	f=0.0000
Excited State	3:	?Spin	-A	1.3928 eV	890.16 nm	f=0.0032

Excited State 4: ?Spin -A 2.0422 eV 607.11 nm f=0.0093
Excited State 5: ?Spin -A 2.1371 eV 580.16 nm f=0.0000
Excited State 6: ?Spin -A 2.3419 eV 529.42 nm f=0.0038
Excited State 7: ?Spin -A 2.5871 eV 479.25 nm f=0.0087
Excited State 8: ?Spin -A 2.7573 eV 449.66 nm f=0.3798
Excited State 9: ?Spin -A 3.0730 eV 403.46 nm f=0.0000
Excited State 10: ?Spin -A 3.1391 eV 394.96 nm f=0.0031

B3LYP TD(nstates=10) /6-311+g(d,p) scrf=(pcm,solvent=water)
Total free energy in solution: with all non-electrostatic terms (a.u.) = -1103.435669
Excitation energies and oscillator strengths:
Excited State 1: ?Spin -A 1.1127 eV 1114.30 nm f=0.1904
Excited State 2: ?Spin -A 1.5403 eV 804.95 nm f=0.0112
Excited State 3: ?Spin -A 1.9033 eV 651.42 nm f=0.0106
Excited State 4: ?Spin -A 2.0479 eV 605.43 nm f=0.0000
Excited State 5: ?Spin -A 2.1573 eV 574.72 nm f=0.0000
Excited State 6: ?Spin -A 2.3106 eV 536.59 nm f=0.0052
Excited State 7: ?Spin -A 2.6403 eV 469.59 nm f=0.3067
Excited State 8: ?Spin -A 2.8368 eV 437.05 nm f=0.2001
Excited State 9: ?Spin -A 3.1093 eV 398.75 nm f=0.0000
Excited State 10: ?Spin -A 3.1714 eV 390.94 nm f=0.0116

Appendix A15. Quercetin radical anion 3′–OH to 3–OH

B3LYP/6-31+g(d,p) gas phase

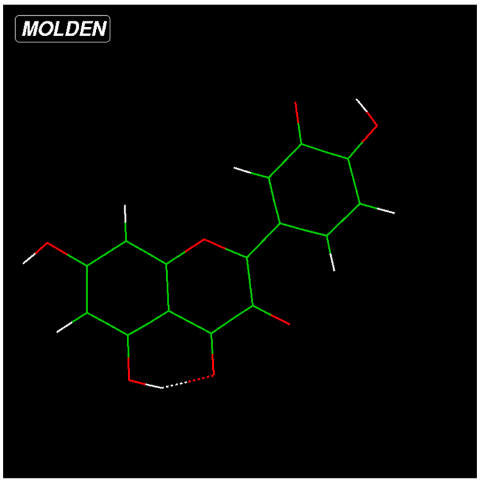

Coordinates:

C,0,-0.0182599685,-0.0000016031,0.0129706061\C,0,-0.0158092435,-0.0000030758,1.4124094781\ C,0,1.1771326706,-0.0000031446,2.1574842479\ C,0,2.4173493605,0.0000026228,1.4508835193\ C,0,2.431307694,0.0000080944,0.0575234078\ C,0,1.2114375919,0.0000044103,-0.6398110731\ C,0,1.1381162877,-0.000005075,3.6017502329\C,0,-0.2087437162,0.0000040549,4.2756956158\C,0,-1.362699303,-0.0000013432,3.4110495067\O,0,-1.2104116801,-0.0000042531,2.0300678575\C,0,-2.7498242863,-0.0000023705,3.7863530613\C,0,-3.108875201,0.0000020799,5.1815903488\C,0,-4.4398219441,0.0000021716,5.5784184915\C,0,-5.436287178,-0.0000013031,4.6047583595\C,0,-5.1357263832,-0.0000050072,3.1705741843\C,0,-3.7725520819,-0.0000055467,2.8088955003\O,0,-6.1582563718,-0.0000083591,2.3993057495\O,0,-6.7484158037,-0.0000010309,4.8794689565\ O,0,2.1970626564,0.0000242158,4.277621604\O,0,-0.2512017749,0.0000024675,5.5288233814\ O,0,3.5707159573,0.000007595,2.1373781732\O,0,1.1880245838,0.0000088643,-2.0136115463\H,0,-0.9484264696,-0.0000028676,-0.541388076\H,0,3.383926872,0.0000138504,-0.4650382603\ H,0,3.2985031496,0.0000013648,3.1120499343\ H,0,2.0952349862,0.0000137964,-2.3448891444\H,0,-2.3112593628,0.0000053864,5.91188442\ H,0,-4.7081560739,0.0000053028,6.6311204539\ H,0,-3.5279776629,-0.0000082845,1.7547254127\H,0,-7.129605635,-0.0000036127,3.9550961463

Thermal correction to Enthalpy= 0.217948
Thermal correction to Gibbs Free Energy= 0.152881
Lowest freq: 89.3482 cm-1

B3LYP TD(nstates=10) /6-311+g(d,p)
HF=-1103.3600907
Excitation energies and oscillator strengths:

Excited State	1:	?Spin	-A	0.9005 eV	1376.87 nm	f=0.0543
Excited State	2:	?Spin	-A	1.6931 eV	732.28 nm	f=0.0000
Excited State	3:	?Spin	-A	1.9754 eV	627.63 nm	f=0.0640

Excited State 4: ?Spin -A 2.1895 eV 566.27 nm f=0.0000
Excited State 5: ?Spin -A 2.2803 eV 543.72 nm f=0.0089
Excited State 6: ?Spin -A 2.5167 eV 492.64 nm f=0.2773
Excited State 7: ?Spin -A 2.8071 eV 441.68 nm f=0.0402
Excited State 8: ?Spin -A 2.8483 eV 435.30 nm f=0.0120
Excited State 9: ?Spin -A 3.0791 eV 402.67 nm f=0.0014
Excited State 10: ?Spin -A 3.0939 eV 400.73 nm f=0.0000

B3LYP TD(nstates=10) /6-311+g(d,p) scrf=(pcm,solvent=water)
Total free energy in solution: with all non-electrostatic terms (a.u.) = -1103.435014
Excitation energies and oscillator strengths:
Excited State 1: ?Spin -A 0.9464 eV 1310.02 nm f=0.0844
Excited State 2: ?Spin -A 1.8910 eV 655.65 nm f=0.0000
Excited State 3: ?Spin -A 2.0180 eV 614.38 nm f=0.0023
Excited State 4: ?Spin -A 2.2365 eV 554.36 nm f=0.2622
Excited State 5: ?Spin -A 2.4234 eV 511.61 nm f=0.0336
Excited State 6: ?Spin -A 2.4549 eV 505.06 nm f=0.0000
Excited State 7: ?Spin -A 2.5514 eV 485.95 nm f=0.0750
Excited State 8: ?Spin -A 2.6631 eV 465.57 nm f=0.1069
Excited State 9: ?Spin -A 3.0474 eV 406.86 nm f=0.0316
Excited State 10: ?Spin -A 3.1669 eV 391.50 nm f=0.0000

Appendix A16. Quercetin radical anion 4′–OH to 3–OH

B3LYP/6-31+g(d,p) gas phase

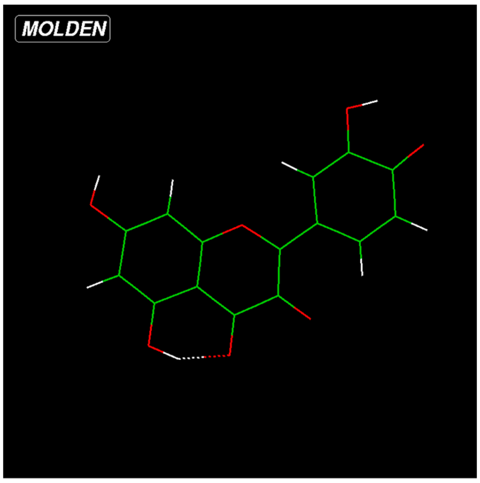

Coordinates:
C,0,0.0022947557,-0.0000125581,-0.0083119787\C,0,0.0030805475,-0.0000005136,1.4289049999\ C,0,1.2749597088,0.0000082534,2.0990964467\C,0,2.4358680711,-0.0000028289,1.3770989989\ C,0,2.456589382,-0.0000263486,-0.0853641172\C,0,1.1734988585,-0.0000198678,-0.7298875635\C,0,-1.2032962912,0.000008966,2.1779906587\O,0,-0.9699663399,0.0000144052,3.5492250275\C,0,-1.9551115843,0.0000087977,4.469504702\C,0,-3.3042354779,-0.0000005056,4.0849662219\C,0,-3.6657815029,-0.0000132213,2.6830949524\C,0,-2.561637532,0.0000030356,1.671211446\C,0,-4.2999794574,-0.0000035445,5.1104802777\C,0,-3.9328368508,-0.0000144682,6.4548866632\C,0,-2.571711501,-0.0000101734,6.7863197332\C,0,-1.5692250006,0.0000045827,5.8151104349\O,0,-5.5971455987,0.0000172047,4.7663559714\O,0,-2.2648682864,-0.0000166739,8.1261064967\O,0,-2.8490137602,-0.000056226,0.4501789392\O,0,-4.872914324,-0.0000569574,2.3286546775\ O,0,3.6674414805,-0.0000012768,1.9572024775\ O,0,3.5846295544,0.0000030676,-0.6630366838\H,0,-0.514608047,0.0000092057,6.0728161236\H,0,-4.6885800426,-0.0000182563,7.2312410623\H,0,-5.6045240055,0.0000732707,3.7543451185\H,0,-1.304529407,-0.0000109523,8.2309252359\H,0,-0.9564604012,-0.0000115168,-0.5093656847\ H,0,1.1564808653,-0.0000200849,-1.816235579\ H,0,1.3227126154,0.0000197837,3.1808600022\ H,0,4.2725007545,-0.0000291147,1.1815410773

Thermal correction to Enthalpy= 0.218440
Thermal correction to Gibbs Free Energy= 0.153480
Lowest freq: 31.3014 cm-1

B3LYP TD(nstates=10) /6-311+g(d,p)
HF=-1103.3734359
Excitation energies and oscillator strengths:

Excited State	1:	?Spin	-A	1.5523 eV	798.69 nm	f=0.0194
Excited State	2:	?Spin	-A	1.7863 eV	694.07 nm	f=0.0000
Excited State	3:	?Spin	-A	2.2246 eV	557.34 nm	f=0.3589

Excited State 4: ?Spin -A 2.4413 eV 507.87 nm f=0.1597
Excited State 5: ?Spin -A 2.5111 eV 493.74 nm f=0.0000
Excited State 6: ?Spin -A 2.8379 eV 436.89 nm f=0.0177
Excited State 7: ?Spin -A 2.9633 eV 418.40 nm f=0.0136
Excited State 8: ?Spin -A 3.0697 eV 403.90 nm f=0.0000
Excited State 9: ?Spin -A 3.1379 eV 395.11 nm f=0.0000
Excited State 10: ?Spin -A 3.1491 eV 393.72 nm f=0.0050

B3LYP TD(nstates=10) /6-311+g(d,p) scrf=(pcm,solvent=water)
Total free energy in solution: with all non-electrostatic terms (a.u.) = -1103.446118
Excitation energies and oscillator strengths:
Excited State 1: ?Spin -A 1.5087 eV 821.81 nm f=0.0725
Excited State 2: ?Spin -A 1.9994 eV 620.11 nm f=0.0000
Excited State 3: ?Spin -A 2.2114 eV 560.66 nm f=0.2336
Excited State 4: ?Spin -A 2.3055 eV 537.78 nm f=0.3432
Excited State 5: ?Spin -A 2.5384 eV 488.44 nm f=0.0465
Excited State 6: ?Spin -A 2.6256 eV 472.21 nm f=0.0000
Excited State 7: ?Spin -A 2.6509 eV 467.71 nm f=0.0095
Excited State 8: ?Spin -A 2.9760 eV 416.62 nm f=0.0338
Excited State 9: ?Spin -A 3.2485 eV 381.67 nm f=0.0000
Excited State 10: ?Spin -A 3.4719 eV 357.11 nm f=0.0213

Appendix A17. Quercetin radical anion 3–OH to 5–OH

B3LYP/6-31+g(d,p) gas phase

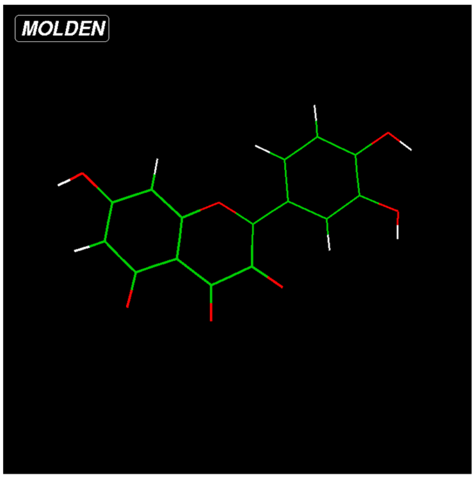

Coordinates:
C,0,-0.024116405,0.0000003917,0.0026664697\ C,0,-0.0074560519,0.0000004835,1.4196278799\ C,0,1.2545985979,0.0000006036,2.0634738528\C,0,2.4208776692,0.0000009864,1.3169641807\C,0,2.3932833229,0.000001066,-0.0849031624\C,0,1.1579995173,0.0000006686,-0.732954355\C,0,-1.2493067258,0.0000003896,2.1749190521\O,0,-2.3465240378,-0.000000291,1.3800251923\C,0,-3.6320360895,-0.0000002318,1.8773207318\C,0,-3.9060008286,0.0000004645,3.2591191426\C,0,-2.806821887,0.0000011312,4.2120045652\C,0,-1.3888744716,0.0000011016,3.6106542159\C,0,-5.3297971096,0.0000005111,3.6737566315\C,0,-6.3103108991,-0.0000002054,2.5914459744\C,0,-5.9481624652,-0.000000894,1.2662184882\C,0,-4.5945640466,-0.0000009275,0.8729793884\O,0,-5.7127891351,0.000001125,4.861680666\O,0,-6.876228631,-0.0000015659,0.2401113656\O,0,-0.3974084805,0.000001768,4.3693219714\O,0,-2.8999781375,0.0000017472,5.4383271086\ O,0,3.6870854008,0.0000009172,1.8832993445\O,0,3.5580176045,0.000001543,-0.8102802756\H,0,-4.3092275952,-0.0000014625,-0.1714573963\H,0,-7.355350526,-0.0000001659,2.894922122\H,0,-7.7579060813,-0.0000014967,0.6351053638\ H,0,-0.9744194016,0.0000002002,-0.516127538\ H,0,1.1386679788,0.0000007636,-1.8185777219\ H,0,1.2798144746,0.0000004575,3.1482016036\ H,0,4.2959077482,0.0000028062,-0.1820821995\ H,0,3.6044707868,0.0000060387,2.8457967402

Thermal correction to Enthalpy= 0.217771
Thermal correction to Gibbs Free Energy= 0.150310
Lowest freq: 28.9929 cm-1

B3LYP TD(nstates=10) /6-311+g(d,p)
HF= -1103.3270752
Excitation energies and oscillator strengths:

Excited State	1:	?Spin	-A	0.9320 eV	1330.24 nm	f=0.0000
Excited State	2:	?Spin	-A	1.0300 eV	1203.74 nm	f=0.0956
Excited State	3:	?Spin	-A	1.8050 eV	686.90 nm	f=0.0000

Excited State 4: ?Spin -A 2.0130 eV 615.91 nm f=0.0360
Excited State 5: ?Spin -A 2.3191 eV 534.62 nm f=0.0000
Excited State 6: ?Spin -A 2.5807 eV 480.43 nm f=0.0223
Excited State 7: ?Spin -A 2.5969 eV 477.43 nm f=0.0000
Excited State 8: ?Spin -A 2.7060 eV 458.18 nm f=0.0432
Excited State 9: ?Spin -A 2.8392 eV 436.68 nm f=0.1231
Excited State 10: ?Spin -A 3.0045 eV 412.66 nm f=0.0000

B3LYP TD(nstates=10) /6-311+g(d,p) scrf=(pcm,solvent=water)
Total free energy in solution: with all non-electrostatic terms (a.u.) = -1103.426401
Excitation energies and oscillator strengths:
Excited State 1: ?Spin -A 1.2572 eV 986.18 nm f=0.1444
Excited State 2: ?Spin -A 1.4598 eV 849.32 nm f=0.0000
Excited State 3: ?Spin -A 1.9849 eV 624.63 nm f=0.0255
Excited State 4: ?Spin -A 2.2664 eV 547.06 nm f=0.0042
Excited State 5: ?Spin -A 2.2928 eV 540.74 nm f=0.0000
Excited State 6: ?Spin -A 2.5754 eV 481.42 nm f=0.1662
Excited State 7: ?Spin -A 2.7251 eV 454.97 nm f=0.1277
Excited State 8: ?Spin -A 2.8842 eV 429.88 nm f=0.0060
Excited State 9: ?Spin -A 2.9067 eV 426.54 nm f=0.0000
Excited State 10: ?Spin -A 3.1158 eV 397.92 nm f=0.0000

Appendix A18. Quercetin radical anion 3–OH to 7–OH

B3LYP/6-31+g(d,p) gas phase

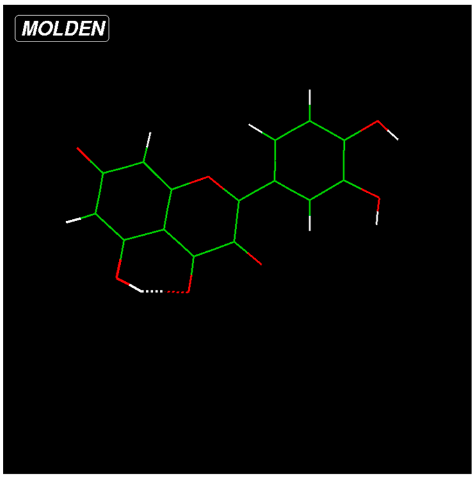

Coordinates:

C,0,0.0047340414,0.0000010217,-0.0140597\C,0,-0.0038209355,0.0000008455,1.4028102137\ C,0,1.24553545,0.0000013017,2.0709478032\C,0,2.4253978283,0.000001886,1.3465674973\C,0,2.4228876172,0.0000020694,-0.0559981323\C,0,1.1997579678,0.0000016169,-0.7273221201\C,0,-1.2622473788,0.0000001671,2.1314686539\O,0,-2.340221986,-0.0000004516,1.3080141053\C,0,-3.6363020749,-0.0000004443,1.7788448787\C,0,-3.8818950596,-0.000000274,3.1751594865\C,0,-2.8221658711,-0.0000037468,4.1212911973\C,0,-1.414206225,-0.0000007007,3.5720564867\C,0,-5.2737452966,0.0000005496,3.5878706531\C,0,-6.2949988313,-0.0000016177,2.6687319756\C,0,-6.0382620491,-0.0000026605,1.2428436421\C,0,-4.6326853961,-0.0000014559,0.8360274855\O,0,-5.5348984568,0.0000064795,4.9143594775\O,0,-6.9539065733,-0.0000041373,0.3810152864\O,0,-0.4349193671,-0.0000007536,4.3487319468\O,0,-3.0002700175,-0.0000070708,5.3705010475\ O,0,3.6808192842,0.0000023932,1.9343415028\O,0,3.5988185954,0.0000026961,-0.7589330976\H,0,-4.4000509198,-0.0000020893,-0.223120733\H,0,-7.3263490613,-0.000001188,3.0049908803\H,0,-4.6436156654,0.00001453,5.3740297528\H,0,-0.9356363265,0.0000006662,-0.5502973359\ H,0,1.200675816,0.0000017553,-1.812938715\ H,0,1.2512032332,0.0000012859,3.1557212171\ H,0,4.3265208453,0.0000029995,-0.1187703669\H,0,3.5837743409,0.000002643,2.8954682097

Thermal correction to Enthalpy= 0.218205

Thermal correction to Gibbs Free Energy= 0.152471

Lowest freq: 31.5131 cm-1

B3LYP TD(nstates=10) /6-311+g(d,p)

HF=- 1103.3565527

Excitation energies and oscillator strengths:

Excited State	1:	?Spin	-A	0.9988 eV	1241.33 nm	f=0.0482
Excited State	2:	?Spin	-A	1.5386 eV	805.82 nm	f=0.0000
Excited State	3:	?Spin	-A	1.6979 eV	730.21 nm	f=0.0000

Excited State 4: ?Spin -A 1.7226 eV 719.73 nm f=0.0602
Excited State 5: ?Spin -A 2.4446 eV 507.17 nm f=0.0015
Excited State 6: ?Spin -A 2.5245 eV 491.13 nm f=0.0387
Excited State 7: ?Spin -A 2.7579 eV 449.56 nm f=0.0000
Excited State 8: ?Spin -A 2.7890 eV 444.55 nm f=0.2150
Excited State 9: ?Spin -A 3.1181 eV 397.63 nm f=0.0460
Excited State 10: ?Spin -A 3.2235 eV 384.62 nm f=0.0219

B3LYP TD(nstates=10) /6-311+g(d,p) scrf=(pcm,solvent=water)
Total free energy in solution: with all non-electrostatic terms (a.u.) = -1103.437261
Excitation energies and oscillator strengths:
Excited State 1: ?Spin -A 1.3674 eV 906.75 nm f=0.0784
Excited State 2: ?Spin -A 1.7975 eV 689.77 nm f=0.0811
Excited State 3: ?Spin -A 1.8602 eV 666.50 nm f=0.0000
Excited State 4: ?Spin -A 2.2014 eV 563.21 nm f=0.0054
Excited State 5: ?Spin -A 2.2661 eV 547.13 nm f=0.0000
Excited State 6: ?Spin -A 2.5030 eV 495.35 nm f=0.1942
Excited State 7: ?Spin -A 2.6824 eV 462.21 nm f=0.1871
Excited State 8: ?Spin -A 2.7929 eV 443.93 nm f=0.0047
Excited State 9: ?Spin -A 3.0901 eV 401.22 nm f=0.0000
Excited State 10: ?Spin -A 3.3356 eV 371.70 nm f=0.0055

Appendix A19. Quercetin radical anion 5–OH to 4'–OH

B3LYP/6-31+g(d,p) gas phase

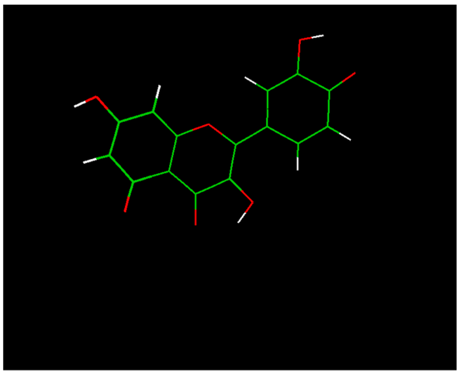

Coordinates:

C,0,0.0270913281,0.,-0.0461771015\C,0,-0.005414375,0.,1.3866306569\ C,0,1.2306419807,0.,2.0974881935\ C,0,2.4177812332,0.,1.4082878238\ C,0,2.4789247197,0.,-0.0535412097\C,0,1.2159309596,0.,-0.7381551285\C,0,-1.2462607324,0.,2.1112643741\ O,0,-1.093112621,0.,3.4748752222\ C,0,-2.1540408927,0.,4.3504680358\ C,0,-3.50270182,0.,3.9048396923\ C,0,-3.7411470282,0.,2.4974512601\ C,0,-2.5411726172,0.,1.6254780944\ C,0,-4.5868815432,0.,4.9190452465\ C,0,-4.1540594678,0.,6.3042172484\ C,0,-2.8234935325,0.,6.6513418947\ C,0,-1.7904464582,0.,5.6864712535\ O,0,-5.7978928445,0.,4.6145960575\ O,0,-2.4055881737,0.,7.9658436934\ O,0,-2.8746279797,0.,0.3276695093\ O,0,-4.8255590658,0.,1.862190567\ O,0,3.6235466392,0.,2.0262902035\ O,0,3.620577943,0.,-0.5998632612\H,0,-0.7492753293,0.,5.9827932868\ H,0,-4.9378739039,0.,7.0582790309\ H,0,-3.8786808587,0.,0.4104160917\ H,0,-3.1885195545,0.,8.5320880998\ H,0,-0.9081515463,0.,-0.589321962\ H,0,1.231022674,0.,-1.8240116849\ H,0,1.2368726422,0.,3.1803517679\ H,0,4.2614791466,0.,1.2780051007

Thermal correction to Enthalpy= 0.218009

Thermal correction to Gibbs Free Energy= 0.152311

Lowest freq: 32.7474 cm-1

B3LYP TD(nstates=10) /6-311+g(d,p)

HF=-1103.4455708

Excitation energies and oscillator strengths:

Excited State	1:	?Spin	-A'	0.9041 eV	1371.40 nm	f=0.1487
Excited State	2:	?Spin	-A"	1.7748 eV	698.58 nm	f=0.0000
Excited State	3:	?Spin	-A'	1.8352 eV	675.57 nm	f=0.0245
Excited State	4:	?Spin	-A'	1.9677 eV	630.11 nm	f=0.0078
Excited State	5:	?Spin	-A'	2.1529 eV	575.90 nm	f=0.0279
Excited State	6:	?Spin	-A"	2.1880 eV	566.67 nm	f=0.0000
Excited State	7:	?Spin	-A'	2.5970 eV	477.42 nm	f=0.1583

Excited State 8: ?Spin -A' 2.7893 eV 444.49 nm f=0.1584
Excited State 9: ?Spin -A'' 3.0666 eV 404.30 nm f=0.0000
Excited State 10: ?Spin -A' 3.3031 eV 375.36 nm f=0.1524

B3LYP TD(nstates=10) /6-311+g(d,p) scrf=(pcm,solvent=water)
Total free energy in solution: with all non-electrostatic terms (a.u.) = -1103.427734
Excitation energies and oscillator strengths:
Excited State 1: ?Spin -A' 0.9041 eV 1371.40 nm f=0.1487
Excited State 2: ?Spin -A'' 1.7748 eV 698.58 nm f=0.0000
Excited State 3: ?Spin -A' 1.8352 eV 675.57 nm f=0.0245
Excited State 4: ?Spin -A' 1.9677 eV 630.11 nm f=0.0078
Excited State 5: ?Spin -A' 2.1529 eV 575.90 nm f=0.0279
Excited State 6: ?Spin -A'' 2.1880 eV 566.67 nm f=0.0000
Excited State 7: ?Spin -A' 2.5970 eV 477.42 nm f=0.1583
Excited State 8: ?Spin -A' 2.7893 eV 444.49 nm f=0.1584
Excited State 9: ?Spin -A'' 3.0666 eV 404.30 nm f=0.0000
Excited State 10: ?Spin -A' 3.3031 eV 375.36 nm f=0.1524

Appendix A20. Quercetin radical anion 5–OH to 7–OH

B3LYP/6-31+g(d,p) gas phase

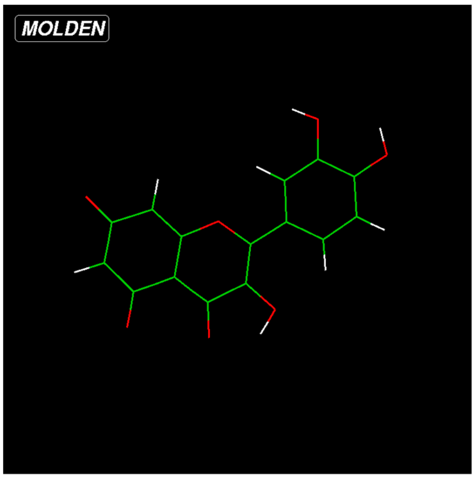

Coordinates:
C,0,0.0250590833,0.0000003435,-0.0577379875\C,0,-0.0177842372,0.0000003875,1.3515910926\C,0,1.2064268437,0.0000007167,2.0543110948\C,0,2.4130755342,0.0000012837,1.3708825976\C,0,2.4483627139,0.000001348,-0.0304455674\C,0,1.2457149378,0.0000008161,-0.733423498\C,0,-1.2760612124,0.0000000708,2.1046038325\O,0,-1.0795259239,0.0000005447,3.4608114083\C,0,-2.1344793664,0.0000004078,4.3555595542\C,0,-3.4949518862,-0.0000001749,3.9364450356\C,0,-3.7429432969,-0.0000009163,2.512487866\C,0,-2.5532889217,-0.0000006744,1.6309894881\C,0,-4.596987471,0.000000065,4.9626942446\C,0,-4.1631157761,0.0000005365,6.325777901\C,0,-2.806239384,0.0000010049,6.7219272831\C,0,-1.7679818355,0.0000009583,5.683294662\O,0,-5.7988750667,-0.0000002961,4.6114750333\O,0,-2.409744811,0.0000014916,7.9361383602\O,0,-2.8825482514,-0.0000013565,0.3177586676\O,0,-4.8356526748,-0.0000011101,1.9050790205\O,0,3.6506855933,0.0000014691,1.9941601731\O,0,3.6440242316,0.0000019212,-0.70163837\H,0,-0.7272245637,0.0000013718,5.9856429842\H,0,-4.9256400192,0.0000006048,7.0976641431\H,0,-3.877221399,-0.0000021942,0.3780523708\H,0,-0.8985687831,0.000000008,-0.6199873198\H,0,1.2774582778,0.0000009009,-1.818533644\ H,0,1.1982949888,0.0000005216,3.1395713838\H,0,4.3554337958,0.0000030868,-0.0438989302\H,0,3.5341843734,0.0000056663,2.9526197113

Thermal correction to Enthalpy=	0.220461
Thermal correction to Gibbs Free Energy=	0.153307

Lowest freq: 22.5841 cm-1

B3LYP TD(nstates=10) /6-311+g(d,p)
HF=-1103.3224478
Excitation energies and oscillator strengths:

Excited State	1:	?Spin	-A	0.2193 eV 5653.23 nm	f=0.0052
Excited State	2:	?Spin	-A	0.6815 eV 1819.40 nm	f=0.0000
Excited State	3:	?Spin	-A	1.1835 eV 1047.60 nm	f=0.0000

Excited State 4: ?Spin -A 1.8129 eV 683.92 nm f=0.0357
Excited State 5: ?Spin -A 2.1115 eV 587.19 nm f=0.0044
Excited State 6: ?Spin -A 2.5662 eV 483.15 nm f=0.0089
Excited State 7: ?Spin -A 2.6220 eV 472.86 nm f=0.0852
Excited State 8: ?Spin -A 2.6590 eV 466.27 nm f=0.0000
Excited State 9: ?Spin -A 2.8097 eV 441.28 nm f=0.1078
Excited State 10: ?Spin -A 2.9073 eV 426.46 nm f=0.0000

B3LYP TD(nstates=10) /6-311+g(d,p) scrf=(pcm,solvent=water)
Total free energy in solution: with all non-electrostatic terms (a.u.) = -1103.412556
Excitation energies and oscillator strengths:
Excited State 1: ?Spin -A 0.4850 eV 2556.31 nm f=0.0074
Excited State 2: ?Spin -A 1.1278 eV 1099.33 nm f=0.0000
Excited State 3: ?Spin -A 1.3338 eV 929.54 nm f=0.1212
Excited State 4: ?Spin -A 1.6334 eV 759.08 nm f=0.0000
Excited State 5: ?Spin -A 2.0489 eV 605.12 nm f=0.0008
Excited State 6: ?Spin -A 2.3325 eV 531.55 nm f=0.0078
Excited State 7: ?Spin -A 2.6145 eV 474.23 nm f=0.0328
Excited State 8: ?Spin -A 2.7707 eV 447.48 nm f=0.0000
Excited State 9: ?Spin -A 2.8954 eV 428.21 nm f=0.0097
Excited State 10: ?Spin -A 3.0732 eV 403.43 nm f=0.0080

Appendix A21. Quercetin radical anion 3′–OH to 7–OH

B3LYP/6-31+g(d,p) gas phase

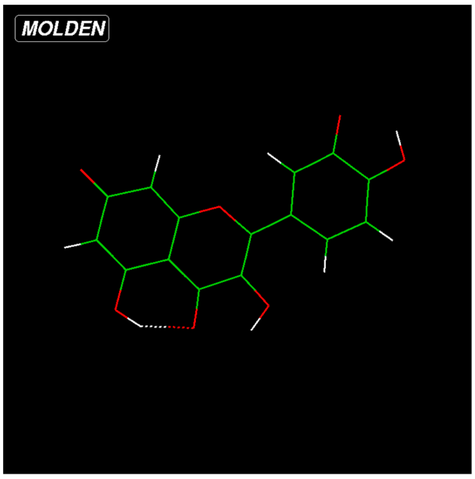

Coordinates:
C,0,0.00676412,0.,-0.0395134414\ C,0,0.019059818,0.,1.3958684442 \C,0,1.2414033872,0.,2.0758843845\ C,0,2.4755751401,0.,1.3688878847\C,0,2.4032741152,0.,-0.0938885621\C,0,1.1889485446,0.,-0.7723633914\ C,0,-1.2353622022,0.,2.1384113738\O,0,-1.046590892,0.,3.4971393508 \C,0,-2.0851427001,0.,4.403879016\ C,0,-3.4319517079,0.,3.9310674699\C,0,-3.6822798828,0.,2.5464973732 \C,0,-2.5251042034,0.,1.656685413\C,0,-4.4904276717,0.,4.9133374473\C,0,-4.202276383,0.,6.2517198169\ C,0,-2.8292570615,0.,6.740630086\ C,0,-1.771549897,0.,5.7371178709\ O,0,-5.7770571391,0.,4.47347475\O,0,-2.5647605341,0.,7.9683990721\O,0,-2.8670690699,0.,0.3453143226\ O,0,-4.8345538287,0.,1.9860936469\ O,0,3.6448128067,0.,1.8706567792\ O,0,3.5921553022,0.,-0.7123529864\ H,0,-0.7400436514,0.,6.0700654955\ H,0,-5.0024278666,0.,6.9840628478\ H,0,-3.8554871352,0.,0.3887553077\ H,0,-5.7507694691,0.,3.4848984321\ H,0,-0.9398985437,0.,-0.5602302402\ H,0,1.1726451999,0.,-1.8580158882\ H,0,1.274344384,0.,3.1575741259\ H,0,4.2257890167,0.,0.0508484937

Thermal correction to Enthalpy= 0.218604
Thermal correction to Gibbs Free Energy= 0.153584
Lowest freq: 19.4202 cm-1

B3LYP TD(nstates=10) /6-311+g(d,p)
HF=-1103.3649958
Excitation energies and oscillator strengths:

Excited State	1:	?Spin	-A'	0.7111 eV	1743.44 nm	f=0.1070
Excited State	2:	?Spin	-A"	1.2168 eV	1018.92 nm	f=0.0000
Excited State	3:	?Spin	-A'	1.2227 eV	1013.98 nm	f=0.0039
Excited State	4:	?Spin	-A'	1.6339 eV	758.81 nm	f=0.0180
Excited State	5:	?Spin	-A"	1.9005 eV	652.37 nm	f=0.0000

Excited State 6: ?Spin -A' 2.2346 eV 554.83 nm f=0.0160
Excited State 7: ?Spin -A' 2.5002 eV 495.89 nm f=0.0021
Excited State 8: ?Spin -A' 2.6796 eV 462.70 nm f=0.1789
Excited State 9: ?Spin -A' 2.8681 eV 432.28 nm f=0.0559
Excited State 10: ?Spin -A" 2.9952 eV 413.94 nm f=0.0000

B3LYP TD(nstates=10) /6-311+g(d,p) scrf=(pcm,solvent=water)
Total free energy in solution: with all non-electrostatic terms (a.u.) = -1103.428206
Excitation energies and oscillator strengths:
Excited State 1: ?Spin -A' 0.8415 eV 1473.29 nm f=0.0945
Excited State 2: ?Spin -A' 1.4793 eV 838.14 nm f=0.0160
Excited State 3: ?Spin -A' 1.6750 eV 740.21 nm f=0.0111
Excited State 4: ?Spin -A" 2.0129 eV 615.96 nm f=0.0000
Excited State 5: ?Spin -A" 2.0214 eV 613.35 nm f=0.0000
Excited State 6: ?Spin -A' 2.3127 eV 536.10 nm f=0.0417
Excited State 7: ?Spin -A' 2.4909 eV 497.75 nm f=0.0411
Excited State 8: ?Spin -A' 2.8365 eV 437.11 nm f=0.1928
Excited State 9: ?Spin -A' 2.9580 eV 419.15 nm f=0.1178
Excited State 10: ?Spin -A" 3.1384 eV 395.06 nm f=0.0000
